# Rational evolution of Cd^2+^-specific DNAzymes with phosphorothioate modified cleavage junction and Cd^2+^ sensing

**DOI:** 10.1093/nar/gkv519

**Published:** 2015-05-18

**Authors:** Po-Jung Jimmy Huang, Juewen Liu

**Affiliations:** Department of Chemistry, Waterloo Institute for Nanotechnology, University of Waterloo, Waterloo, Ontario N2L 3G1, Canada

## Abstract

*In vitro* selection of RNA-cleaving DNAzymes is a powerful method for isolating metal-specific DNA. A few successful examples are known, but it is still difficult to target some thiophilic metals such as Cd^2+^ due to limited functional groups in DNA. While using modified bases expands the chemical functionality of DNA, a single phosphorothioate modification might boost its affinity for thiophilic metals without complicating the selection process or using bases that are not commercially available. In this work, the first such *in vitro* selection for Cd^2+^ is reported. After using a blocking DNA and negative selections to rationally direct the library outcome, a highly specific DNAzyme with only 12 nucleotides in the catalytic loop is isolated. This DNAzyme has a cleavage rate of 0.12 min^−1^ with 10 μM Cd^2+^ at pH 6.0. The *R*_p_ form of the substrate is cleaved ∼100-fold faster than the *S*_p_ form. The DNAzyme is most active with Cd^2+^ and its selectivity against Zn^2+^ is over 100 000-fold. Its application in detecting Cd^2+^ is also demonstrated. The idea of introducing single modifications in the fixed region expands the scope of DNA/metal interactions with minimal perturbation of DNA structure and property.

## INTRODUCTION

Being a negatively charged polymer with four heterocyclic nucleobases, DNA is quite versatile in metal binding using electrostatic and coordination interactions (1–5), and this interaction is a key topic in bioinorganic, analytical and medicinal chemistry (5–8). To obtain metal-specific DNA, *in vitro* selection of RNA-cleaving DNAzymes is a powerful method (7,9–16). For example, DNAzymes with high specificity for lead (17–19), uranium (20,21) and lanthanide ions have been reported (22–24). These metals are either hard or borderline Lewis acids, interacting favorably with the phosphate of DNA. On the other hand, success is limited for selections against many other metals, possibly due to the limited chemical functionality in DNA (25). To overcome this problem, modified DNA bases (e.g. with imidazole) were introduced (26–29). However, two limitations confine their broader applications. First, these modified bases are not commercially available. Second, such selections are technically demanding, and few such DNAzymes were reported.

We reason that a single modification near the substrate RNA cleavage site might bring sufficient affinity for metal binding. For example, phosphorothioate (PS) refers to replacing one of the non-bridging phosphate oxygen atoms by sulfur (30,31). PS might be a good choice for binding thiophilic metals, and this is supported by previous ribozyme studies. Many ribozymes (and some DNAzymes) have Mg^2+^-dependent activities. After the PS modification, these enzymes became less active with Mg^2+^ but are activated by soft metals such as Mn^2+^ or Cd^2+^ (32–35). Using PS DNA to build metal binding sites offers the following advantages. First, PS is commercially available at a low cost (∼$3 per modification). In fact, PS-modified DNA is a widely used tool in chemical biology (36) and materials science (37–41). Second, the PS modification is made in a fixed (instead of randomized) region of the DNA library and does not complicate *in vitro* selection.

Cadmium is a highly toxic metal known for its carcinogenicity (42). In the last century, discharge of Cd^2+^ has increased tremendously, leading to contamination of water and agriculture products such as rice (43). Most reported optical sensors for Cd^2+^ are based on fluorescent chelators (44). However, they often require organic solvents and are strongly interfered by Zn^2+^ (sometimes also by Ca^2+^, Hg^2+^ and Pb^2+^), suggesting challenges associated with rational ligand design. Herein, we performed Cd^2+^-dependent DNAzyme selections using a PS-modified library. This is the first effort of rationally introducing a PS for *in vitro* selection. Since each PS produces two diastereomers, we also developed a DNAzyme-based method for chiral separation. Finally, a biosensor for Cd^2+^ was developed with ultrahigh sensitivity and specificity.

## MATERIALS AND METHODS

### Chemicals

The DNAs for selection (Supplementary Table S1) and sensing were purchased from Integrated DNA Technologies (Coralville, IA, USA). The other DNAs were from Eurofins (Huntsville, AL, USA; Supplementary Table S2). The metal salts were from Sigma-Aldrich at the highest available purity. Tris(hydroxymethyl)aminomethane (Tris), 2-(N-morpholino)ethanesulfonic acid (MES), 2-[4-(2-hydroxyethyl)piperazin-1-yl]ethanesulfonic acid (HEPES), ethylenediaminetetraacetic acid (EDTA), NaCl and ammonium acetate were from Mandel Scientific (Guelph, Ontario, Canada). SsoFast EvaGreen supermix was from Bio-Rad. T4-DNA ligase, dNTP mix, Taq DNA polymerase and DNA ladder were from New England Biolabs.

### *In vitro* selection

Supplementary Figure S1 shows our selection scheme. The initial library was prepared by ligating Lib-FAM (0.2 nmol) and Lib-rA* (0.3 nmol) with a splint DNA (0.3 nmol) using T4 ligase following the vendor's protocol. The ligated DNA was purified with 10% denaturing polyacrylamide gel electrophoresis (dPAGE) and extracted from the gel with buffer B (1 mM EDTA, 10 mM Tris-HCl, pH 7.0). After ethanol precipitation, the library was re-suspended in 60 μl buffer C (50 mM MES, pH 6.0, 25 mM NaCl) and used for the first round of selection. For each subsequent round, the library was generated from polymerase chain reaction (PCR). For blocked selection, before each selection step, the library was annealed with 150 pmol of each of the two blocking DNAs to inactivate the Ce13-related sequences (note Ce13 is a previously reported DNAzyme that dominated the final library of our initial selection in this work) (22). After incubating with Cd^2+^ at room temperature (see Supplementary Table S3 for incubation time and metal concentration), the reaction was quenched with 8-M urea and the cleaved product was purified by 10% dPAGE. The selected DNA was extracted from the gel, desalted with a Sep-Pak C18 column (Waters) and then suspended in 70 μl HEPES buffer (5 mM, pH 7.5). Two PCR steps were used to amplify the selected DNA. In PCR1, P1 and P2 primers were used. In PCR2, P3 and P4 were used as described previously (22). For negative selections, the library was treated with a metal mixture containing Zn^2+^, Pb^2+^ and Cu^2+^ (20 μM each). The uncleaved oligonucleotides were harvested for a positive selection with Cd^2+^.

### Sequencing

Three DNA sequencing experiments were performed. For each one, the PCR1 product was cloned using the TA-TOPO cloning kit and transformed into Efficiency DH5α competent cells following the vendor's protocol. The plasmid DNA was extracted and purified using DirectPrep 96 miniprep kit (QIAGEN). The extracted DNA was submitted to TCAG DNA Sequencing Facility (Toronto, ON, Canada).

### Enzyme assays

Gel-based assays were performed with carboxyfluorescein (FAM)-labeled PS substrate (0.7 μM) and enzyme (1.1 μM) annealed in buffer C. A final of 10 μM Cd^2+^ (or other metals/concentrations) was added to initiate the cleavage reaction. The products were separated on a dPAGE gel and analyzed using a ChemiDoc MP imaging system (Bio-Rad). All assays were run at least in duplicate.

### HPLC separation of diastereomers

An Agilent 1260 Infinity Quaternary HPLC system with an Inspire C18 column (5 μm, 250 mm × 4.6 mm, Dikma) was used for the diastereomers separation. The HPLC separation was carried out based on previously published protocols (30). The 5′-half of the substrate containing the PS modification was injected at 20 μl (100 μM) with a flow rate of 1 ml/min. The column was heated to 45°C. Two solvents were prepared for eluting the sample: solvent A = 0.1 M NH_4_OAc; solvent B = 20% 0.1 M NH_4_OAc and 80% CH_3_CN. From time 0 to 10 min, 97% solvent A and 3% solvent B were used. From 10 min to 90 min, the solvent A was gradually decreased to 90%, while solvent B increased to 10%.

For the subsequent ligation reaction, HPLC-5′, HPLC-3′ and splint DNA were mixed at 1:1.5:1.5 ratio in buffer (10 mM MgCl_2_, 50 mM Tris-HCl, pH 7.5). The mixture was annealed at 95 °C for 1 min followed by slow cooling to room temperature. The T4 ligation protocol provided by the vendor was followed. The ligated DNA product was purified with 15% dPAGE at 650 V for 1.5 h, and DNA was extracted from the gel with buffer (1 mM EDTA, 10 mM Tris-HCl, pH 7.0). The extracted DNA was further desalted using a Sep-Pak C18 cartridge (Waters). The purified DNA was then lyophilized overnight and re-suspended in 5-mM HEPES (pH 7.5) for further analysis.

### DNAzyme-based chiral separation

The FAM-labeled PS substrate (1 μM) was annealed with 17E or BN-Cd16 (3 μM) in buffer D (50 mM MOPS, pH 7.5, 25 mM NaCl) or buffer C, respectively. MgCl_2_ (10 mM) was added to the 17E sample (overnight), while CdCl_2_ (10 μM) was added to the BN-Cd16 sample (1 h). Both samples were then desalted with Sep-Pak columns, and the uncleaved substrate was separated by 10% dPAGE. After another desalting step, the purified substrate was re-suspended in 5 mM HEPES (pH7.5) and the DNA concentration was determined by Nanodrop 1000 (Thermo).

### Biosensor assays

The sensing kinetics were measured in a 96-well plate using a microplate reader (M3, SpectraMax). The sensor complex was formed by annealing the FAM-labeled PS substrate (after 17E treatment) and the quencher-labeled enzyme (molar ratio = 1:1.5) in buffer C. The final sensor concentration was 50 nM in 1 mM HEPES (pH 7.5, 100 μl each well). One microliter metal ion was added to initiate cleavage and the signaling kinetics was monitored (*Ex* = 485 nm; *Em* = 520 nm).

### Detecting Cd^2+^ in extracted rice matrix

White rice was from a local supermarket and ground into fine powders. The rice powder (500 mg) was loaded in a Pyrex tube and HCl (100 mM, 1 ml) was added. After cooking at 95 °C for 3 h, NaOH (100 mM, 1 ml) was added to neutralize the sample. After centrifugation, the supernatant was collected. For detection, 2 μl of the extracted sample with various concentrations Cd^2+^ was added into 98 μl sensor.

## RESULTS AND DISCUSSION

### Direct selections with a PS-modified cleavage junction

**Figure 1.**
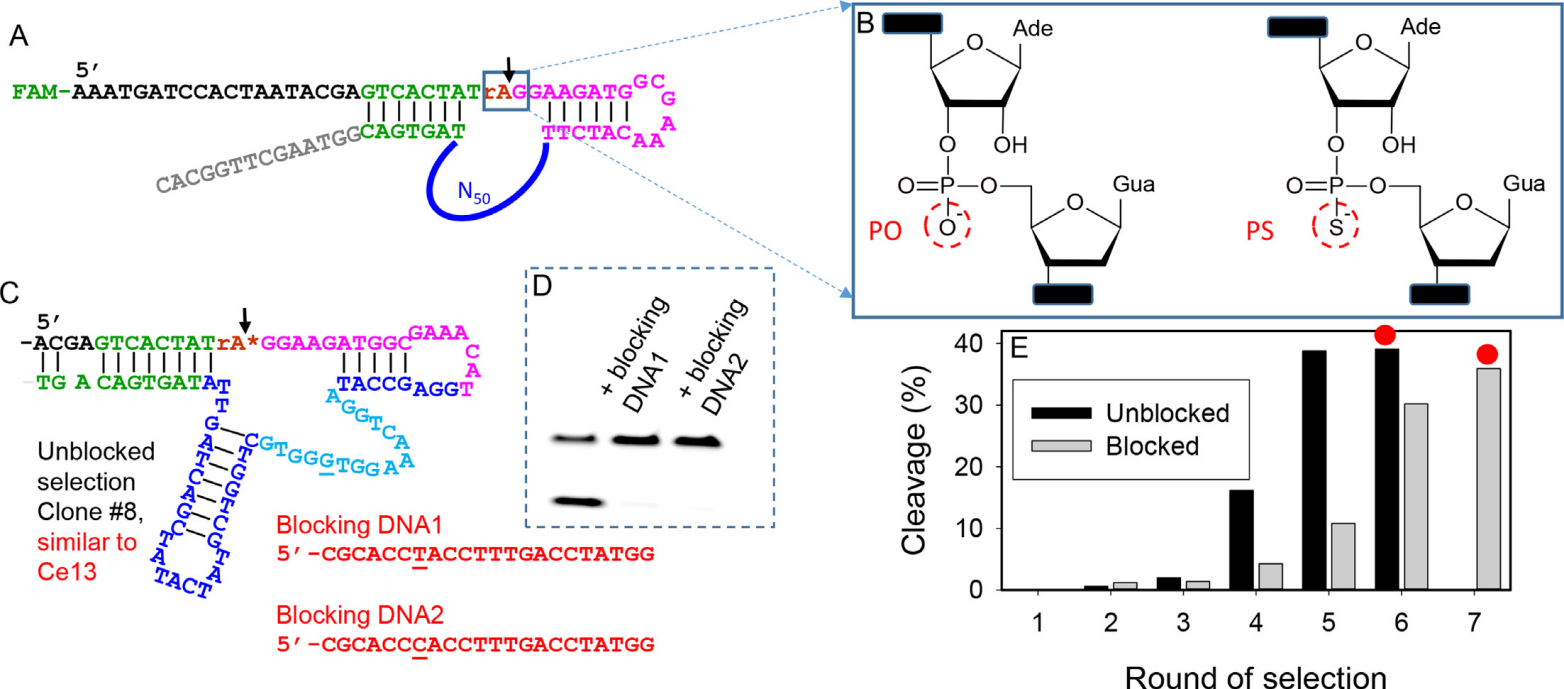
(**A**) The library sequence for the PS DNAzyme selection. The structure of the cleavage junction (rAG) is shown in (**B**), where rA denotes ribo-adenosine. Instead of the normal PO, all the selections in this work used the PS linkage. (**C**) A representative sequence from the direct selection, where the blue/cyan nucleotides are from the randomized N_50_ region. The asterisk at the cleavage site represents the PS. This sequence is similar to Ce13, a previously reported DNAzyme. Two blocking sequences are complementary to the cyan region and they differ only by one base (underlined). (**D**) Cleavage of the Ce13 DNAzyme is inhibited by the blocking DNAs. (**E**) Progress of the direct (unblocked) and blocked selections. The red dots indicate the rounds for DNA sequencing.

To isolate Cd^2+^-specific DNA, *in vitro* selection was carried out with a library containing 50 random nucleotides (N_50_, ∼10^14^ random sequences; Figure 1A). The cleavage site is indicated by the arrowhead at the single RNA (rA) position. This scissile bond is ∼1-million-fold less stable compared to the rest DNA linkages (45). A PS modification was introduced at this cleavage junction (Figure 1B) to increase affinity toward thiophilic Cd^2+^. All the previous selections used only the normal phosphate (PO) linkage (17,22,46).

In each round, Cd^2+^ was added to induce cleavage. The cleaved oligonucleotides were harvested by gel electrophoresis and amplified by PCR to seed the next round of selection. Saturated activity was obtained after five rounds (Figure 1E, black bars), yielding ∼35% cleavage. The round 6 library was cloned and sequenced. Interestingly, 34 out of the 35 obtained sequences are similar to the Ce13 DNAzyme, which was first isolated in a lanthanide-dependent selection (22). A representative sequence (Figure 1C) shows a hairpin (in blue) and a large loop (in cyan) that constitute the catalytic core. Each individual clone may differ in the hairpin but the loop sequence is highly conserved (see Supplementary Table S4 for sequence alignment). Since Ce13 is active with Cd^2+^ using a PS-modified substrate (35), it is not surprising that it was isolated again. Note that Ce13 with the normal substrate (i.e. no PS modification) is active only with lanthanides and Pb^2+^. If the cleavage junction was not PS-modified, it is unlikely that Ce13 can be isolated. This result also suggests that Ce13 is a preferred (or easy-to-obtain) solution for Cd^2+^-dependent PS RNA cleavage. However, this DNAzyme is not specific for Cd^2+^ (e.g. also active with Pb^2+^, Cu^2+^, Hg^2+^ and Ce^3+^).

### Blocked selections

**Figure 2.**
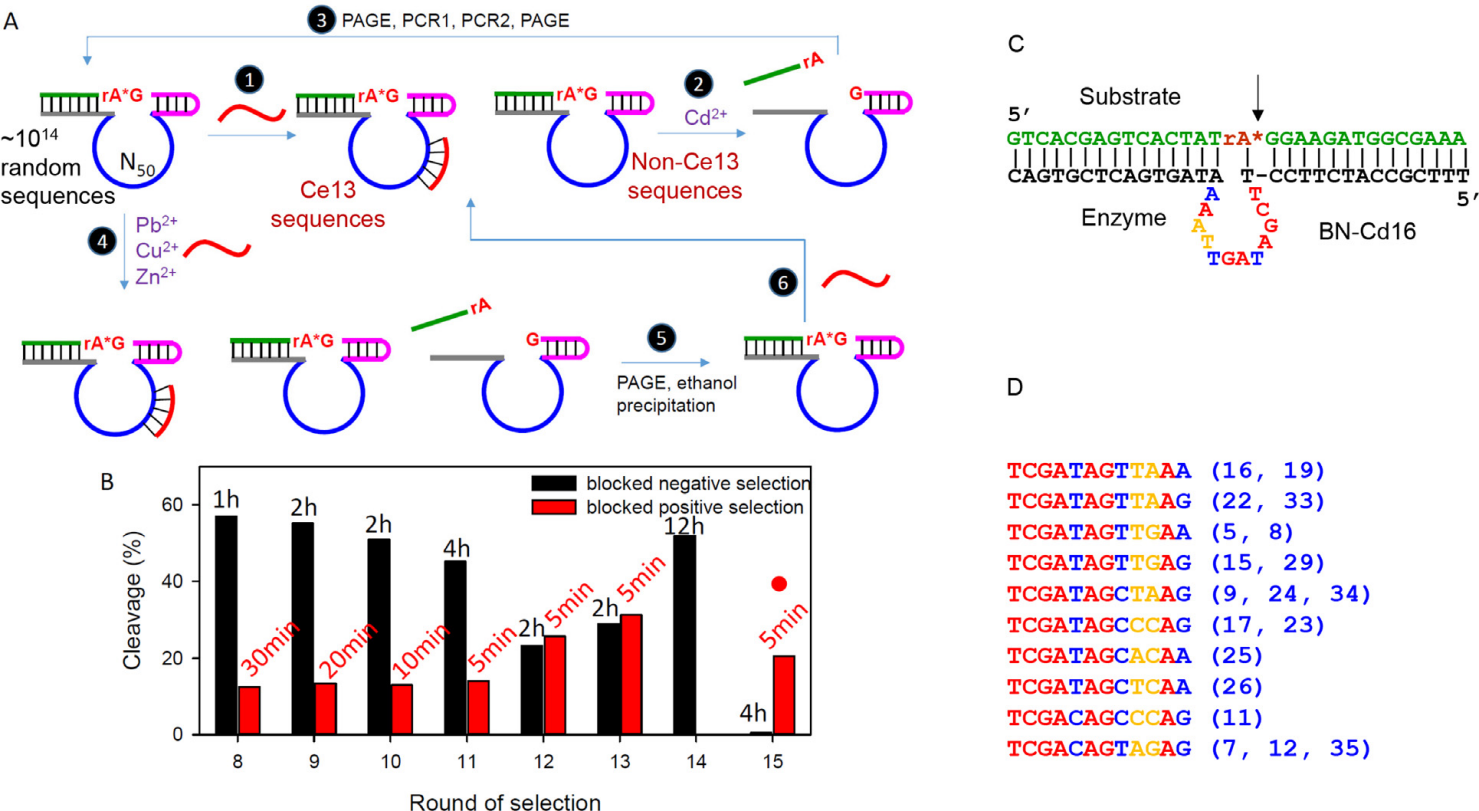
(**A**) A scheme for the blocked selection and with negative selection. The blockers are intended to eliminate the Ce13 sequences. Steps 1, 2 and 3 consist of a completely blocked selection cycle. From round 8, negative selections were carried out with a metal mixture. In this case, the uncleaved oligonucleotides were collected (steps 4, 5 and 6) and then reacted with Cd^2+^ for the positive selection (steps 2 and 3). (**B**) Selection progress from round 8 of the blocked selection. For each round, both positive and negative selections were carried out. The round 15 library was sequenced. (**C**) A trans-cleaving DNAzyme derived from BN-Cd16. (**D**) Alignment of the enzyme loop for sequences similar to BN-Cd16. Nucleotides in red are absolutely conserved, in blue can be purine or pyrimidine substituted and in yellow are variable. The clone numbers are in the parentheses. The color coding matches that in (C).

To isolate new DNAzymes, we decided to re-select. A few methods (e.g. mutagenic PCR) might increase sequence diversity and reduce the Ce13 sequence. Here we developed a new method based on the property of Ce13. We noticed that the conserved sequence of Ce13 is quite long (e.g. the 15 nucleotides in cyan in Figure 1C). Only one of the nucleotides (marked by underline) may change from G to A (22). We hypothesized that the library might be evolved against this sequence by using blocking DNA complementary to these conserved nucleotides. Two blocking DNAs were designed in Figure 1C, and they can completely inactivate the Ce13 DNAzyme (Figure 1D). In our new selection scheme (Figure 2A), an excess amount of blocking DNAs (150 pmol) was first hybridized with the library to inactivate the Ce13 sequences (step 1). Then Cd^2+^ was added (step 2) and the cleaved oligonucleotides were amplified (step 3). The progress was slightly slower and it took seven rounds to reach activity plateau (Figure 1B, gray bars). This is probably due to suppression of the highly active Ce13 population. The round 7 library was sequenced and the Ce13 variants were indeed eliminated (see Supplementary Table S5 for sequence alignment). This enriched library however has a high sequence diversity, suggesting that many solutions for Cd^2+^-dependent cleavage are available. After treating the round 7 library with a metal mixture (Pb^2+^, Cu^2+^ and Zn^2+^, 20 μM each), nearly 60% cleavage occurred after 1 h, indicating this library still lacked specificity for Cd^2+^.

### Blocked negative selections

These competing metals are all thiophilic, which may explain their activity. Aside from thiophilicity, they may differ in other aspects, such as size, coordination chemistry and the *p*K_a_ value of the bound water. These differences might be picked up by some DNA sequences. Under this assumption, we further evolved the library using negative selection. After incubating with a metal mixture (Pb^2+^, Zn^2+^ and Cu^2+^; step 4, Figure 2A), the cleaved oligonucleotides were discarded and the remaining uncleaved library was harvested (step 5). Then the library was incubated with Cd^2+^ for the positive selection (step 2). To achieve high specificity, stringent conditions were used for the negative selection by extending reaction time (Figure 2B, black bars). The activity of the competing metals went down from 58% cleavage in 1 h in round 8 to ∼30% in 2 h in round 13. After that, we did a 12-h incubation followed by a 4-h incubation. At the end (round 15), we barely observed any cleavage with the metal mixture, suggesting a significant selectivity improvement.

To ensure high activity, the reaction time was shortened for the positive Cd^2+^-dependent selections. In the last four rounds, only 5 min was allowed and ∼20% cleavage was consistently achieved (Figure 2B, red bars). At round 15, since both the negative and positive activities were optimized, this library was sequenced.

### DNAzyme secondary structure analysis

Out of the 37 sequences (Supplementary Table S6), 19 are aligned to a single family, and a representative sequence is clone #16. Supplementary Figure S2 shows the Mfold predicted structure of clone #16 (47), and its *trans*-cleaving structure is shown in Figure 2C, where the redundant nucleotides are removed. The enzyme loop is very small, containing only 12 nucleotides. This loop sequence is well aligned (Figure 2D): the nucleotides in red are highly conserved, in blue can be changed from purine to purine or from pyrimidine to pyrimidine, while the two yellow nucleotides are more variable. Overall, this appears to be a well-defined new DNAzyme.

### High Cd^2+^ specificity

**Figure 3.**
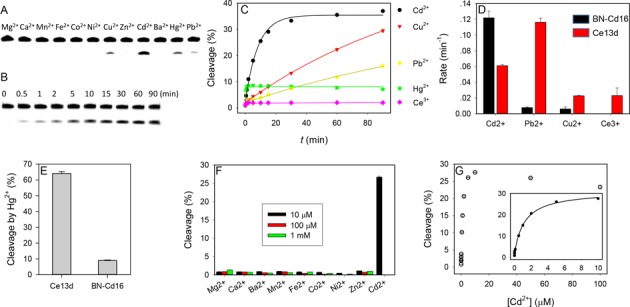
Biochemical characterization of the BN-Cd16 DNAzyme. Gel images of BN-Cd16 with the PS substrate reacting (**A**) in the presence of 10 μM various metals after 10 min incubation, and (**B**) with 10 μM Cd^2+^ as a function of time. (**C**) Kinetics of the PS substrate cleavage by BN-Cd16 with different metal ions (10 μM). (**D**) Comparison of rate of cleavage of BN-Cd16 and Ce13d with the PS substrate. (**E**) Comparison of the fraction of cleavage by Hg^2+^ (10 μM) with these two DNAzymes. (**F**) Cleavage percentage with three concentrations of various competing metals. (**G**) Fraction of cleavage after 15 min as a function of Cd^2+^ concentration. All assays were run in 50 mM MES buffer (pH 6.0) with 25 mM NaCl.

A few sequences in Figure 2D were tested, and they all have similar Cd^2+^-dependent activity (Supplementary Figure S3), confirming the sequence alignment. The #16 sequence was chosen for further studies (named BN-Cd16 or Cd16 in short). Its metal specificity was first measured with 10 μM divalent metal ions (Figure 3A), and Cd^2+^ indeed shows the best cleavage. Moderate activity was observed with Cu^2+^, Pb^2+^ and Hg^2+^, while none of the other metal ions produced any cleavage up to 1 mM (Figure 3F). In particular, the Cd^2+^ selectivity over Zn^2+^ was more than 100 000-fold based on their cleavage rates. Therefore, BN-Cd16 solves the challenging problem of separating Cd^2+^ and Zn^2+^.

Next, we measured the kinetics of BN-Cd16 with the active metal ions, and a gel image with Cd^2+^ is shown in Figure 3B. The cleavage rate with 10-μM Cd^2+^ is 0.12 min^−1^, 15-fold higher than that with Cu^2+^ and 20-fold higher than Pb^2+^ (Figure 3D, black bars). Hg^2+^ produced an interesting cleavage kinetic profile, showing ∼8% cleavage only in the first half minute. Hg^2+^ can cleave PS RNA even in the absence of any DNAzyme due to its extremely strong thiophilicity (48).

For comparison, we also measured the cleavage rate of Ce13 (Figure 3D, red bars), where all the four metals showed significant activity (see Supplementary Figure S4 for DNAzyme sequence). Since the Hg^2+^ rate cannot be accurately measured, the final cleavage yield is compared (Figure 3E). Ce13 produced ∼8-fold more cleavage. Taken together, BN-Cd16 is highly selective for Cd^2+^ and it represents a significant improvement over Ce13. For practical Cd^2+^ detection, we are likely to deal with low nM transition metals, where selectivity for Cd^2+^ will be even better. We next studied the effect of Cd^2+^ concentration (Figure 3G). The highest activity was observed with 10 μM Cd^2+^, and the apparent *K*_d_ is estimated to be 1.2 μM Cd^2+^ (inset of Figure 3G).

In addition to this most abundant family, a few other sequences were also tested. For example, BN-Cd13 (three similar sequences found in the library) is quite active (Supplementary Figure S5A), but not selective (Supplementary Figure S5B). BN-Cd04 has very low activity (Supplementary Figure S5A). BN-Cd18 has poor selectivity (Supplementary Figure S6). BN-Cd40 is quite selective (Supplementary Figure S6) but very slow (Supplementary Figure S7). Overall, BN-Cd16 is an optimal sequence both in terms of activity and specificity for Cd^2+^.

### Stereochemistry

For all these assays, Cd^2+^ cleaved no more than 35% of the substrate. Even after increasing enzyme concentration and reaction time, cleavage was still less than 50% (Supplementary Figure S8). This is much lower than most DNAzymes, where over 80% cleavage can be achieved.

**Figure 4.**
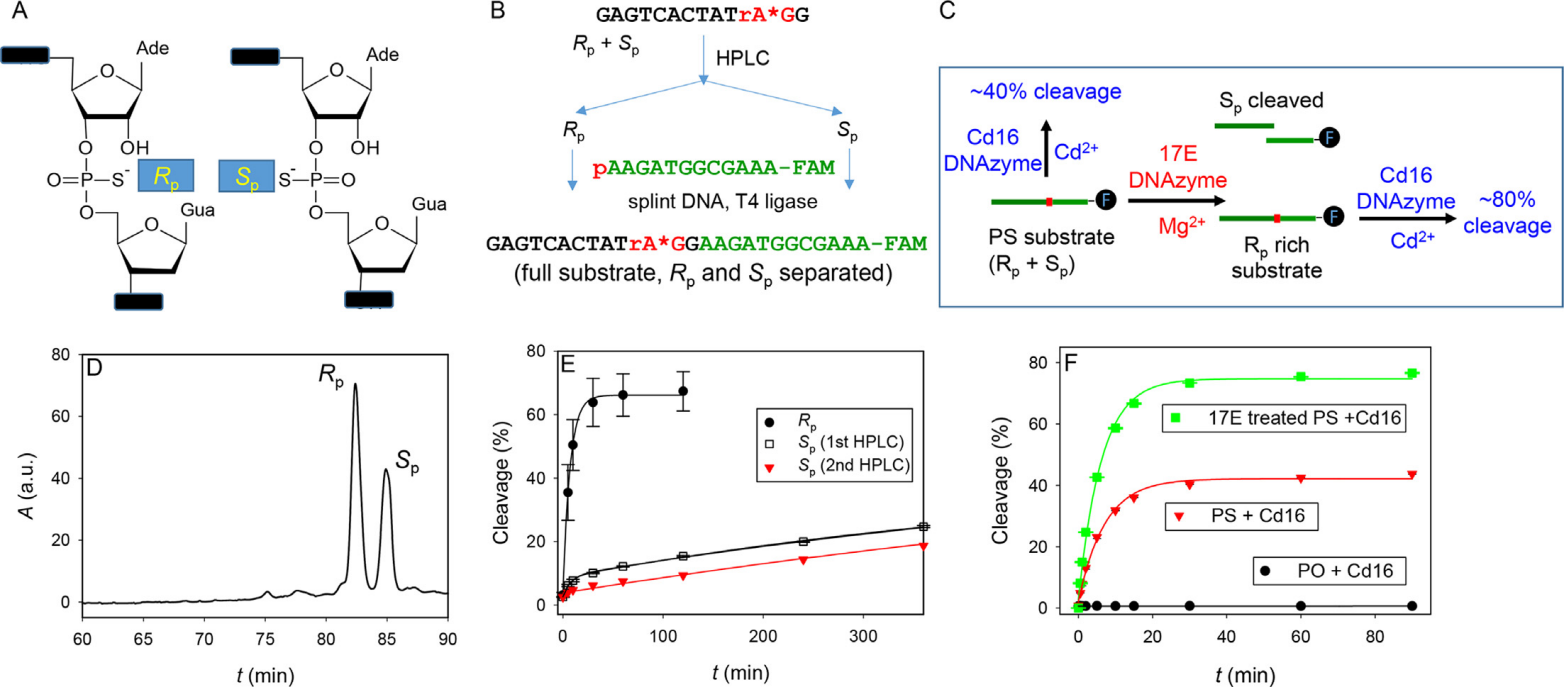
(**A**) The structures of the two PS diastereomers at the cleavage junction. The BN-Cd16 DNAzyme is active only with the *R*_p_ isomer. (**B**) A scheme of HPLC separation followed by ligation to obtain the two diastereomers of the substrate. (**C**) The scheme of experiment design for treating the PS substrate to remove the *S*_p_ population by the 17E DNAzyme and to increase reaction yield. (**D**) An HPLC trace of the separation. (**E**) Kinetics of the *R*_p_ and *S*_p_ substrate cleaved by the BN-Cd16 and Cd^2+^. The *S*_p_ fragment was purified twice to improve purity and data from both are shown. (**F**) Kinetics of PO, PS and treated PS substrate cleavage by the BN-Cd16 DNAzyme in the presence of 10 μM Cd^2+^.

Introducing a PS modification results in two diastereomers at the phosphorus center (*R*_p_ and *S*_p_; Figure 4A). These two diastereomers were studied in ribozymes (32,49,50) and DNAzymes (51,52). Most enzymes use Mg^2+^, which likes oxygen-based ligands. When the pro-*R*_p_ oxygen was replaced by sulfur, the Mg^2+^-dependent activity was nearly abolished (>100-fold slower, e.g. the HDV ribozyme (49) and the hammerhead ribozyme (32)). This activity can often be rescued by thiophilic metals such as Cd^2+^ or Mn^2+^. When the pro-*S*_p_ oxygen was replaced, the effect is much smaller (e.g. ∼5-fold). This indicates that these enzymes use the pro-*R*_p_ oxygen to bind Mg^2+^.

During our *in vitro* selection and assays, these two isomers were not separated. It is likely that only one isomer is active. Since our substrate is quite long (30 mer) and has an FAM fluorophore, we separated only the 5′-half of the substrate containing the PS center and then ligated it with the other half of the substrate bearing a 5′-phosphate and 3′-FAM (Figure 4B) (53). The HPLC separation trace is shown in Figure 4D, where two well-resolved peaks were obtained. The *R*_p_ form is eluted first followed by the *S*_p_ (30), and this has been confirmed by snake venom phosphodiesterase I digestion (Supplementary Figure S9). These two diastereomers were then separately hybridized with the BN-Cd16 DNAzyme and their activities in the presence of Cd^2+^ were measured (Figure 4E). The *R*_p_ substrate has a rate of 0.15 min^−1^, while the *S*_p_ one has two rates: 0.13 min^−1^ and 0.0013 min^−1^. It appears that the *S*_p_ substrate might still contain a small fraction of *R*_p_, and the *R*_p_ form is ∼100-fold faster than the *S*_p_ form. This rate difference is typical for the RNA-cleaving enzymes (49). We then performed a second HPLC purification on the *S*_p_ fragment (Supplementary Figure S10), and this time the cleavage from the fast fraction dropped by ∼6%, while the rate of the slower fragment remained the same (Figure 4E, red triangles). This study has assigned the active form of the substrate and explained the low cleavage yield for the non-separated substrate.

### DNAzyme-based chiral separation

**Figure 5.**
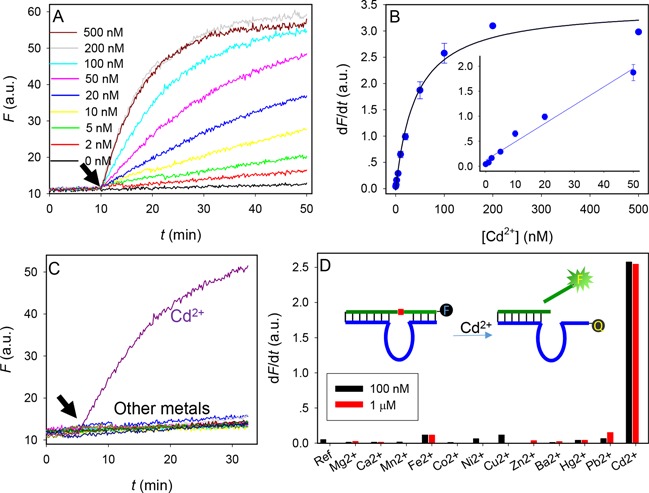
(**A**) Kinetics of sensor fluorescence enhancement with various concentrations of Cd^2+^. The arrowhead points the time of Cd^2+^ addition. (**B**) Initial rate of fluorescence enhancement (from 1 to 10 min after adding Cd^2+^) as a function of Cd^2+^ concentration. Inset: the linear response at low Cd^2+^ concentrations. (**C**) Sensor response to 100 nM of various metals. The list of other metals is in (**D**). (D) Sensor selectivity quantified at two metal concentrations. Inset: a scheme showing the sensor design.

The above method requires both HPLC purification and a ligation step, limiting the yield of products. Since most previously reported DNAzymes employ Mg^2+^ as the metal cofactor and cleave the *S*_p_ form more efficiently, we also developed a DNAzyme-based method for chiral separation. 17E is a well-characterized and Mg^2+^-dependent DNAzyme (11,19,46,54). Since 17E shares the same substrate sequence as the current BN-Cd16 DNAzyme, we reacted our PS substrate with 17E in the presence of 10 mM Mg^2+^. In 90 min, ∼40% cleavage was achieved (data not shown). The uncleaved PS substrate (after the 17E treatment) was isolated after gel electrophoresis and hybridized with the BN-Cd16 DNAzyme. Upon adding Cd^2+^, ∼80% cleavage was achieved (Figure 5A, green dots), which is significantly higher than the untreated PS substrate (Figure 5A, red triangles). The rate of cleavage (0.16 min^−1^) is similar to that of the untreated substrate (0.12 min^−1^), and also similar to the above purified *R*_p_ form. Therefore, the same species is responsible for the cleavage before and after the 17E DNAzyme treatment. Our result implies that the 17E treatment selectively removed the *S*_p_ isomer. The remaining *R*_p_ isomer active with BN-Cd16/Cd^2+^ was thus enriched (Figure 4C). This study adds another example of using nucleic acid based enzymes for chiral separation (55). We also performed mass spectrometry to identify the cleavage product, confirming that cleavage takes place at the PS linkage, yielding a 5′-hydroxy- and 2′,3′-cyclic phosphorothioate product (Supplementary Figure S11).

The 10-23 DNAzyme is a similar Mg^2+^-dependent DNAzyme that was selected together with 17E (46). The 10-23 DNAzyme uses the pro-*R*_p_ oxygen to bind Mg^2+^. Since the 10-23 DNAzyme is a variant of 17E (56), this also supports 17E cleaving the *S*_p_ isomer (52). In preparing our sensor (*vide infra*), we purified 3-nmol substrate in one run, which can be readily scaled up.

In a separate experiment, we also measured the activity of the PO substrate with BN-Cd16 and Cd^2+^, and no cleavage was observed (Figure 4F, black dots). This highlights the importance of the PS. In fact, BN-Cd16 cannot cleave the PO substrate by any tested metals (Supplementary Figure S12), which also explains its high metal specificity (e.g. strong oxygen effect).

### Cd^2+^ turning over

Since Cd^2+^ has relatively strong affinity with sulfur, one question is whether Cd^2+^ can turnover multiple DNAzymes or is it sequestered after each reaction. To test this, we used 5-μM DNAzyme complex and 0.2 μM Cd^2+^. The cleavage fraction was quantified in terms of turnover numbers (Supplementary Figure S13), and multiple turnover is indeed possible. Each turnover takes ∼50 min, and this can be accelerated by using more Cd^2+^. This turnover property might be useful for improving sensitivity for detecting Cd^2+^. While many previous papers have established the multiple turnover ability of DNAzymes for cleaving substrate strands using the same enzyme (46), for the purpose of metal detection, using Cd^2+^ to turnover multiple cleavage events is more relevant (20).

### A Cd^2+^ sensing beacon

To test this DNAzyme for Cd^2+^ detection, a biosensor was designed. We labeled the 5′-end of the substrate with an FAM fluorophore and the 3′-end of the enzyme with a dark quencher (inset of Figure 5D, and Supplementary Figure S14A for sequences). In the hybridized complex, the fluorescence was quenched. When Cd^2+^ was added, a concentration-dependent fluorescence enhancement was observed (Figure 5A). At high Cd^2+^ concentrations, most fluorescence increase took place in the first few minutes. We measured the slope of these kinetic traces from minutes 1 to 10 after adding Cd^2+^ (Figure 5B). The data in the first minute were filtered to eliminate potential Hg^2+^ interference (Supplementary Figure S14B). The detection limit is 1.1 nM Cd^2+^ based on 3σ/slope calculation (inset). The U.S. Environmental Protection Agency (EPA) maximal contamination level in drinking water is 5 μg/l (45 nM) Cd^2+^, which is right in the middle of our dynamic range. We next measured the sensor response to other metal ions (Figure 5C and D), and only Cd^2+^ showed an obvious signal increase. To improve sensitivity, the substrate was first treated with 17E to remove the *S*_p_ population. Without the 17E treatment, the amount of fluorescence enhancement was ∼40% lower (Supplementary Figure S15).

### Detecting Cd^2+^ in rice

**Figure 6.**
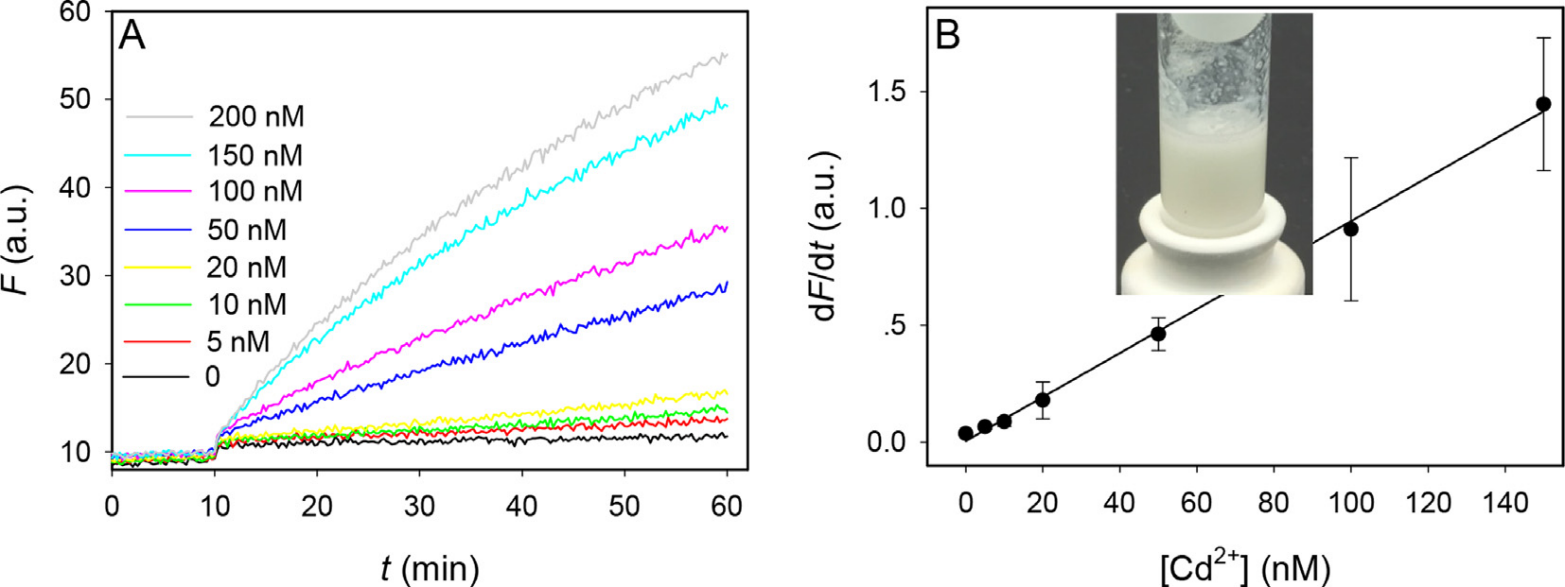
Sensing Cd in rice. (**A**) Sensor response kinetics to various concentrations of Cd^2+^ in rice extracts. The rice samples were added at 10 min. (**B**) The slope of sensor signal increase as a function of Cd^2+^ concentration. Inset is a photograph showing ground rice after heat digestion with acid.

Finally, we aim to test whether our sensor works for rice. The World Health Organization (WHO) has set the limit to be 0.4-mg/kg polished rice grain (i.e. 0.4 ppm). We digested ground rice powder with acid under heating. The digested sample (inset of Figure 6B) was neutralized by base and then diluted 50 times into our sensor solution. After considering the dilution, the Cd^2+^ concentration is 17.8 nM at the toxic limit. The kinetics of the sensor response was monitored (Figure 6A), and the detection limit was 1.6 nM Cd^2+^, which is >10-fold lower than the WHO limit. This proof-of-concept experiment supports the feasibility of using this sensor for rice.

## CONCLUSIONS

In summary, we developed a new method for constructing metal binding sites in DNA by introducing a single PS modification. With this design, three *in vitro* selections were performed to isolate DNA sequences specific for Cd^2+^. The selection outcome was rationally directed by adding blocking DNA and negative selections. This is the first attempt on DNAzyme selection with the PS modification. Compared to selections using modified bases, this single PS does not complicate PCR. For example, the protocol of selection is identical to the normal PO selections and the only difference is that one of the PCR primers contains the PS modification. The resulting DNAzyme is highly selective for Cd^2+^ with over 100 000-fold lower activity with Zn^2+^. Since the PS modification introduces a chiral center, we identified that the *R*_p_ stereoisomer is the active one while the *S*_p_ one is essentially inactive. This DNAzyme was engineered into a highly sensitive biosensor with a detection limit of 1.1-nM Cd^2+^ in buffer and 1.6 nM in rice extract.

## SUPPLEMENTARY DATA

Supplementary Data are available at NAR Online.

SUPPLEMENTARY DATA
