# Supplementary material for: Rational evolution of Cd2+-specific DNAzymes with phosphorothioate modified cleavage junction and Cd2+ sensing
Source: Nucleic Acids Res. 2015 May 18;43(12):6125–33. doi: 10.1093/nar/gkv519 (PMC4499143; doi:10.1093/nar/gkv519)
Supplement: SUPPLEMENTARY DATA [file supp_43_12_6125__index.html]

Rational evolution of Cd2+-specific DNAzymes with phosphorothioate modified cleavage junction and Cd2+ sensing — Rational evolution of Cd2+-specific DNAzymes with phosphorothioate modified cleavage junction and Cd2+ sensing — SUPPLEMENTARY DATA 

# Rational evolution of Cd2+-specific DNAzymes with phosphorothioate modified cleavage junction and Cd2+ sensing

## SUPPLEMENTARY DATA

- SUPPLEMENTARY DATA
