# Supplementary material for: Rational evolution of Cd2+-specific DNAzymes with phosphorothioate modified cleavage junction and Cd2+ sensing
Source: Nucleic Acids Res. 2015 May 18;43(12):6125–33. doi: 10.1093/nar/gkv519 (PMC4499143; doi:10.1093/nar/gkv519)
Supplement: SUPPLEMENTARY DATA [file supp_gkv519_nar-00773-f-2015-File008.pdf]

Supplementary Data

Rational evolution of Cd^{2+}-specific DNAzymes with phosphorothioate modified cleavage junction and Cd^{2+} sensing

Po-Jung Jimmy Huang and Juewen Liu*

Department of Chemistry, Waterloo Institute for Nanotechnology, University of Waterloo, Waterloo, Ontario, Canada, N2L 3G1

Email: liujw@uwaterloo.ca

Table S1. DNA sequences related to in vitro selection in this work. The cleavage site ribo-adenine is denoted by rA, FAM = carboxyfluorescein, iSp18 is an 18-atom hexa-ethyleneglycol spacer. The 5′ of the Lib-rA* DNA is phosphorylated (denoted by the p) for the ligation reaction. The star (*) denotes for phosphorothioate modification.

**DNA Name**	**Sequence and modifications** (from 5′ to 3′)
Lib-FAM	pGGCGAAACATCTT$\mathbf{N_{50}}$TAGTGACGGTAAGCTTGGCAC-**FAM**
Lib-rA*	5'-AATACGAGTCACTAT**rA***GGAAGAT
Splint DNA	5′-AAGATGTTTCGCCATCTTCCTATAGTCCACCACCA
P1 primer	5′-GTGCCAAGCTTACCG
P2 primer	5′-CTGCAGAATTCTAATACGAGTCACTATAGGAAGATGGCGAAACA
P3 primer	5′-**FAM**-AAATGATCCACTAATACGACTCACTAT**rA***GG
P4 primer	5′-AACAACAACAAC-**iSp18**-GTGCCAAGCTTACCG
Blocking DNA1	CGCACCTACCTTTGACCTATGG
Blocking DNA2	CGCACCCACCTTTGACCTATGG

Table S2. The other DNA sequences used in this study. BHQ denotes for Black Hole Quencher® 1, and p denotes for phosphorylation. Most of these sequences are to test the activity of individual clones from the blocked negative selection.

**DNA Name**	**Sequence and modifications** (from 5′ to 3′)
BN-Cd13	CGC CAT CTT CAA TTC GAT AGA GTC CAC GTC TAC AGG AAT GTG GGA AAT AGT GAC TCG TGA
BN-Cd11	TTT CGC CAT CTT CCT TCG ACA GCC CAG ATA GTG ACT CGT GAC
BN-Cd16	TTT CGC CAT CTT CCT TCG ATA GTT AAA ATA GTG ACT CGT GAC
BN-Cd23	TTT CGC CAT CTT CCT TCG ATA GCC CAG ATA GTG ACT CGT GAC
BN-Cd22	TTT CGC CAT CTT TCT TCG ATA GTT AAG ATA GTG ACT CGT GAC
BN-Cd04	TTT CGC CAT CTT GAA ACG CAC GAA GAA TAG TGA CTC GTG AC
BN-Cd40	TTT CGC CAT CTA ACA GGA AAC ACT TTA GTG ACT CGT GAC

BN-Cd18	CGC CAT CTT TAC CCA AAA GGA AGG TTT TCT ATT TTT AGA AAC ACA GGA GTA GTG ACT CGT
PS-Sub	GTC ACG AGT CAC TAT rA*GG AAG ATG GCG AAA-FAM
PO-Sub	GTC ACG AGT CAC TAT rAGG AAG ATG GCG AAA-FAM
Ce13d	TTTC GCC ATA GGT CAA AGG TGG GTG CGA GTT TTT ACT CGT TAT AGT GAC TCG T
17E	TTT CG CCA TCT TCT CCG AGC CGG TCG AAA TAG TGA CTC GTG AC
FAM-Sub	FAM-AGT CACTAT rA*GG AAG ATG GCG AAC
Q-BN-Cd16	GTT CGC CAT CTT CCT TCG ATA GTT AAA ATA GTG ACT-BHQ
HPLC-splint	AAA AAA AAA ATT TCG CCA TCT TCC TAT AGT GAC TC
HPLC-5′	GAGTCACTATrA*GG
HPLC-3′	pAAGATGGCGAAA-FAM

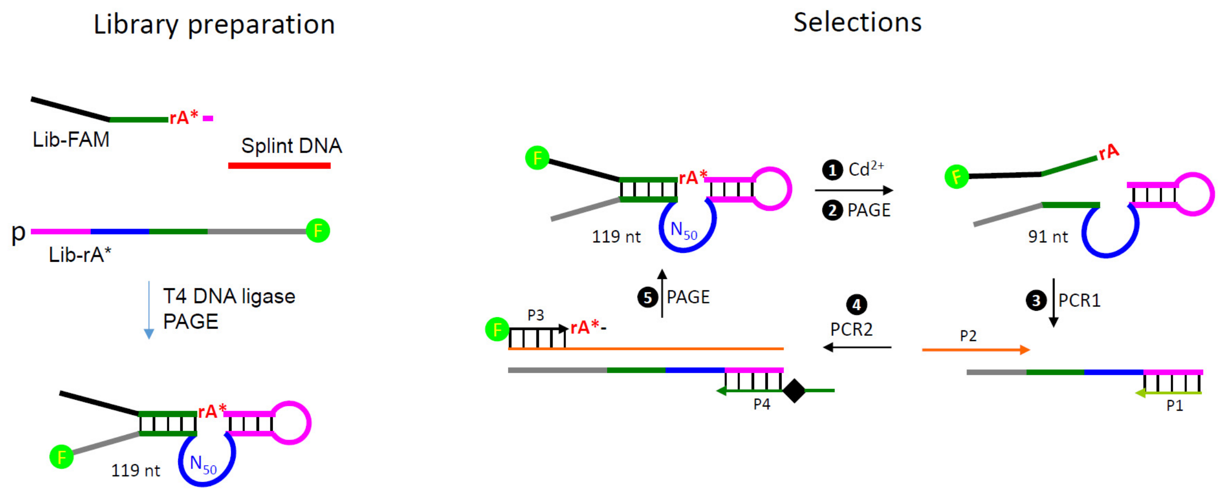

Figure S1. A scheme of direct in vitro selection of Cd^{2+}-dependent PS-modified DNAzymes. The initial library was prepared by a ligation reaction (left). The initial library contains an N50 randomized region and a single RNA linkage (rA) as the cleavage site. The library sequence is shown in Figure 1A of the main paper. A PS modification denoted by the star is incorporated at the cleavage site. The cleaved sequences in the presence of Cd^{2+} (step 1) are harvested after denaturing polyacrylamide gel electrophoresis (PAGE, step 2). The incubation time and metal concentration of each round of selection are shown in Table S3. After two rounds of PCR the full-length single-stranded library is re-generated and the positive strand is isolated after another PAGE

step. In the first PCR, two unmodified primers were used to bring the library back to the full length. In the second PCR, two modified primers were used to produce two strands of DNA with unequal lengths. The primer sequences are shown in Table S1. Finally, the shorter stand bearing the rA and FAM fluorophore is harvested by PAGE to seed the next round of selection. Two variations have been introduced to this protocol by using the blocking DNAs and also using negative selection as described in the Materials and Methods part. This is the basic protocol for the direct selection.

Table S3. In vitro selection conditions in this work. A total of 3 selections were carried out.

Selection 1. Direct selection		
**Round**	**[Cd^{2+}] (µM)**	**Incubation time (min)**
1	50	60
2	50	60
3	50	60
4	50	60
5	50	30
6	50	15
Selection 2. Blocked selection		
**Round**	**[Cd^{2+}] (µM)**	**Incubation time (min)**
1	50	60
2	50	60
3	50	60
4	50	60
5	50	60
6	50	40
7	50	40
Selection 3. Blocked selection with negative selections		
**Round**	**[Zn^{2+}, Cu^{2+}, Pb^{2+}] (-) or [Cd^{2+}] (+) (µM)**	**Incubation time (min)**
8 (-)	50	60

8 (+)	50	30
9 (-)	50	120
9 (+)	50	20
10 (-)	20	120
10 (+)	50	10
11 (-)	20	240
11 (+)	50	5
12 (-)	10	120
12 (+)	50	5
13 (-)	10	120
13 (+)	50	5
14 (-)	10	1440
14 (+)	-	-
15 (-)	10	240
15 (+)	50	5

Table S4. Sequence alignment of the direct selection using the PS library and Cd^{2+}. Sequences aligned together with Ce13 (in green). The cleavage site adenine is marked in red (only marked in the first sequence, all the rest are aligned at this position), and the conserved loop sequence in blue (only shown in the first sequence and the subsequent ones are identical in this region). Only the last sequence (UNBlkCd39) cannot be aligned. The * symbol means fully conserved nucleotides and the dots mean very similar (highly conserved) nucleotides.

```
UNBlkCd07      CTGCAGAATTCTAATACGAGTCACTAT-AGGAAGATGGCGAAACATCTGGGAGCCATAGG 59
UNBlkCd34      CTGCAGAATTCTAATACGAGTCACTAT-AGGAAGATGGCGAAACATCTGGGAGCCATAGG 59
UNBlkCd14      CTGCAGAATTCTAATACGAGTCACTAT-AGGAAGTTGGCGAAACATTTCG-AGCCATAGG 58
UNBlkCd04      CTGCAGAATTCTAATACGAGTCACTAT-AGGAAGATGGCGAAACAT---GGAGCCATAGG 56
UNBlkCd08      CTGCAGAATTCTAATACGAGTCACTAT-AGGAAGATGGCGAAACATCATGGAGCCATAGG 59
Ce13           CTGCAGAATTCTAATACGAGTCACTAT-AGGAAGATGGCGAAACATCTTGGAGCCATAGG 59
UNBlkCd23      CTGCAGAATTCTAATACGAGTCACTAT-AGGAAGATGGCGAAACAT---GGAGCCATAGG 56
UNBlkCd30      CTGCAGAATTCTAATACGAGTCACTAT-AGGAAGATGGCGAAACAT---GGAGCCATAGG 56
UNBlkCd22      CTGCAGAATTCTAATACGAGTCACTAT-AGGAAGATGGCGAAACAT---TGAGCCATAGG 56
UNBlkCd05      CTGCAGAATTCTAATACGAGTCACTAT-AGGAAGATGGCGAAACAT---GGAGCCATAGG 56
UNBlkCd21      CTGCAGAATTCTAATACGAGTCACTAT-AGGAAGATGGCGAAACAT---GGAGCCATAGG 56
UNBlkCd03      CTGCAGAATTCTAATACGAGTCACTATAAGGAAGATGGCGAAACAT---GGAGCCATAGG 57
UNBlkCd36      CTGCAGAATTCTAATACGAGTCACTAT-AGGAAGATGGCGAAACAT---GGAGCCATAGG 56
UNBlkCd15      CTGCAGAATTCTAATACGAGTCACTAT-AGGAAGATGGCGAAACAT---GGAGCCATAGG 56
```

```
UNBlkCd11       CTGCAGAATTCTAATACGAGTCACTAT-AGGAAGATGGCGAAACAT---GGAGCCATAGG 56
UNBlkCd10       CTGCAGAATTCTAATACGAGTCACTAT-AGGAAGATGGCGAAACAT---GGAGCCATAGG 56
UNBlkCd09       CTGCAGAATTCTAATACGAGTCACTAT-AGGAAGATGGCGAAACAT---GGAGCCATAGG 56
UNBlkCd06       CTGCAGAATTCTAATACGAGTCACTAT-AGGAAGATGGCGAAACAT---GGAGCCATAGG 56
UNBlkCd17       CTGCAGAATTCTAATACGAGTCACTAT-AGGAAGATGGCGAAACAT---GGAGCCATAGG 56
UNBlkCd37       CTGCAGAATTCTAATACGAGTCACTAT-AGGAAGATGGCGAAACAT---GGAGCCATAGG 56
UNBlkCd40       CTGCAGAATTCTAATACGAGTCACTAT-AGGAAGATGGCGAAACAT---GGAGCCATAGG 56
UNBlkCd35       CTGCAGAATTCTAATACGAGTCACTAT-AGGAAGATGGCGAAACAT---GGAGCCAGAGG 56
UNBlkCd02       CTGCAGAATTCTAATACGAGTCACTAT-AGGAAGATGGCGAAACAT---GGAGCCATAGG 56
UNBlkCd38       CTGCAGAATTCTAATACGAGTCACTAT-AGGAAGATGGCGAAACAT---GGAGCCATAGG 56
UNBlkCd26       CTGCAGAATTCTAATACGAGTCACTAT-AGGAAGATGGCGAAACAT---GGAGCCATAGG 56
UNBlkCd25       CTGCAGAATTCTAATACGAGTCACTAT-AGGAAGATGGCGAAACAT---GGAGCCATAGG 56
UNBlkCd19       CTGCAGAATTCTAATACGAGTCACTAT-AGGAAGATGGCGAAACAT---GGAGCCATAGG 56
UNBlkCd12       CTGCAGAATTCTAATACGAGTCACTAT-AGGAAGATGGCGAAACAT---GGAGCCATAGG 56
UNBlkCd28       CTGCAGAATTCTAATACGAGTCACTAT-AGGAAGATGGCGAAACAT---GGAGCCATAGG 56
UNBlkCd27       CTGCAGAATTCTAATACGAGTCACTAT-AGGAAGATGGCGAAACAT---GGAGCCATAGG 56
UNBlkCd29       CTGCAGAATTCTAATACGAGTCACTAT-AGGAAGATGGCGAAACAT---G-AGCCATAGG 55
UNBlkCd33       CTGCAGAATTCTAATACGAGTCACTAT-AGGAAGATGGCGAAACAT---GAAGCCATAGG 56
UNBlkCd13       CTGCAGAATTCTAATACGAGTCACTAT-AGGAAGATGGCGAAACAT---GGAGCCATAGG 56
UNBlkCd24       CTGCAGAATTCTAATACGAGTCACTAT-AGGAAGATGGCGAAACAT---GGAGCCATAGG 56
UNBlkCd20       CTGCAGAATTCTAATACGAGTCACTAT-AGGAAGATGGCGAAACATCTTTACAAAAAAAC 59
UNBlkCd39       CTGCAGAATTCTAATACGAGTCACTAT-AGGAAGATGGCGAAACAT--CTTTACTAAGGA 57
                *************************** ******:***********      .. * ..

UNBlkCd07       TCAAA-GGTGGGTGCG-GTCG---TTTAT--CGACTAG-TTATAATGACGGTAAGCTTGG 111
UNBlkCd34       TCAAA-GGTGGGTGCGTGTCG---TATAT--CGACTAA-GTATAGTGACGGTAAGCTTGG 112
UNBlkCd14       TCAAA-GGTGGGTGCGGGTCG---TATAT--CGACTAAGTTATAGTGACGGTAAGCTTGG 112
UNBlkCd04       TCAAA-GGTTGGTGCTGGTCGCATTATAT--CGACT-AGTTATAGTGACGGTAAGCTTGG 112
UNBlkCd08       TCAAA-GGTGGGTGCTG-TCGCATCATAT--CGACT--GTTATAGTAACGGTAAGCTTGG 113
Ce13            TCAAA-GGTAGGTGCGGGTCGTATCATAT--CGACT-AGTTATAGTGACGGTAAGCTTGG 115
UNBlkCd23       TCAAA-GGTGGGTGCTAGTCGTATCATAT--CGACT-AG-TATAGTGACGGTAAGCTTGG 111
UNBlkCd30       TCAAA-GGTGGGTGCTAGTCGTATCATAT--CGACT-AGTTATAGTGACGGTAAGCTTGG 112
UNBlkCd22       TCAAA-GGTGGGTGCTAGTCGTATCATAC--CGACT-AGTTATAGTGACGGTAAGCTTGG 112
UNBlkCd05       TCAAA-GGTGGGTGCGAGTCGTATCATAT--CGACG-AG-TATAGTGACGGTAAGCTTGG 111
UNBlkCd21       TCAAA-GGTGGGTGCGAGTCGTATCATAT--CGACG-AG-TATAGTGACGGTAAGCTTGG 111
UNBlkCd03       TCAAA-GGTGGGTGCGAGTCGTATCATAT--CGACG-AGTTATAGTGACGGTAAGCTTGG 113
UNBlkCd36       TCAAA-GGTGGGTGCGAGTCGTATCATAT--CGACG-AGTTATAGTGACGGTAAGCTTGG 112
UNBlkCd15       TCAAA-GGTGGGTGCGAGTCGTATCATAT--CGACG-AGTTATAGTGACGGTAAGCTTGG 112
UNBlkCd11       TCAAA-GGTGGGTGCGAGTCGTATCATAT--CGACG-AGTTATAGTGACGGTAAGCTTGG 112
UNBlkCd10       TCAAA-GGTGGGTGCGAGTCGTATCATAT--CGACG-AGTTATAGTGACGGTAAGCTTGG 112
UNBlkCd09       TCAAA-GGTGGGTGCGAGTCGTATCATAT--CGACG-AGTTATAGTGACGGTAAGCTTGG 112
UNBlkCd06       TCAAA-GGTGGGTGCGAGTCGTATCATAT--CGACG-AGTTATAGTGACGGTAAGCTTGG 112
UNBlkCd17       TCAAA-GGTGGGTGCGAGTCGTATCATAC--CGACG-AGTTATAGTGACGGTAAGCTTGG 112
UNBlkCd37       TCAAA-GGTGGGTGCGAGTCGTATCATAC--CGACG-AGTTATAGTGACGGTAAGCTTGG 112
UNBlkCd40       TCAAA-GGTGGGTGCGAGTCGTATCATAT--CGGCG-AGTTATAGTGACGGTAAGCTTGG 112
UNBlkCd35       TCAAA-GGTGGGTGCGAGTCGTATCATAT--CGACG-AGTTATAGTGACGGTAAGCTTGG 112
UNBlkCd02       TCGAA-GGTGGGCGCGAGTCGTATCATAT--CGACG-AGTTATAGTGACGGTAAGCTTGG 112
UNBlkCd38       TCAAA-GGTGGGTGCGAGTCGTATCACAT--CGACG-AGTTATAGTGACGGTAAGCTTGG 112
UNBlkCd26       TCAAA-GGTGGGTGCGAGTCGTATCATAT--CGACGAAGTTATAGTGACGGTAAGCTTGG 113
UNBlkCd25       TCAAA-GGTGGGTGCGAGTCGTATCATAT--CGACGAAGTTATAGTGACGGTAAGCTTGG 113
UNBlkCd19       TCAAA-GGTGGGTGCGAGTCGAATCATGT--CGACGAAGTTATAGTGACGGTAAGCTTGG 113
UNBlkCd12       TCAAA-GGTGGGTGCGAGTCGTATCTTAT--CGACGAAGTTATAGTGGCGGTAAGCTTGG 113
UNBlkCd28       TCAAA-GGTGGGTGCGAGTCGTATCTTAT--CGACGAAGTTATAGTGACGGTAAGCTTGG 113
UNBlkCd27       TCAAA-GGTGGGTGTG-GTCGTATCTTAT--CGAC-CAGTAATAGTGACGGTAAGCTTGG 111
UNBlkCd29       TCAAA-GGTGGGTGCGAGTCGTATCATAT--CGACGAAGTAATAGTGACGGTAAGCTTGG 112
UNBlkCd33       TCAAA-GGTGGGTGCGAGTCGTATCATAT--CAACT-CG-TATAGTGACGGTAAGCTTGG 111
UNBlkCd13       TCAAA-GGTGGGTGCGTGTCGTATCATAT--CGACTAAG-TATAGTGACGGTAAGCTTGG 112
UNBlkCd24       TCAAA-GGTGGGTGCGTGTCG-ATCTAAT--CGACTAAGTTATAGTGACGGTAAGCTTGG 112
UNBlkCd20       TCAAA-AGTTGGCCCTTCCCGCATTTAAG--TTTTCGGAAGATAGTGACGGTAAGCTTGG 116
UNBlkCd39       GCAAATAGCGAGAAACCGGCGAAACTAGAGGCGATCTGG-GTTAGTGACGGTAAGCTTGG 116
                 *.** .*  .*       **    : .             :**.*..************
```

Table S5. Sequence alignment of the blocked selection (without negative selection) using the two blocking sequences. Only four sequences (07, 26, 18, 23) belong to the BN-Cd16 family.

```
BlkCd07         CTGCAGAATTCTAATACGAGTCACTATAGGAAGATGGCGAAACATCTTCATTCG------ 54
BlkCd26         CTGCAGAATTCTAATACGAGTCACTATAGGAAGATGGCGAAACATCTTCATTCG------ 54
BlkCd03         CTGCAGAATTCTAATACGAGTCACTATAGGAAGATGGCGAAACATCTTATCACG------ 54
BlkCd10         CTGCAGAATTCTAATACGAGTCACTATAGGAAGATGGCGAAACATCTTAAGTCAT----- 55
BlkCd31         CTGCAGAATTCTAATACGAGTCACTATAGGAAGATGGCGAAACATCTTCACTAGT----- 55
BlkCd18         CTGCAGAATTCTAATACGAGTCACTATAGGAAGATGGCGAAACATCTTAGTCAGA----- 55
BlkCd23         CTGCAGAATTCTAATACGAGTCACTATAGGAAGATGGCGAAACATTTTT-AGAGC----- 54
BlkCd19         CTGCAGAATTCTAATACGAGTCACTATAGGAAGATGGCGAAACATCTTT---TAG----- 52
BlkCd16         CTGCAGAATTCTAATACGAGTCACTATAGGAAGATGGCGAAACATCTTT--ACGT----- 53
BlkCd01         CTGCAGAATTCTAATACGAGTCACTATAGGAAGATGGCGAAACATCTTTACTAGGCA--- 57
BlkCd27         CTGCAGAATTCTAATACGAGTCACTATAGGAAGATGGCGAAACATCTTTACTAGCCT--- 57
BlkCd15         CTGCAGAATTCTAATACGAGTCACTATAGGAAGATGGCGAAACATCTTTACTAGCAC--- 57
BlkCd21         CTGCAGAATTCTAATACGAGTCACTATAGGAAGATGGCGAAACATCTTTACTAACG---- 56
BlkCd02         CTGCAGAATTCTAATACGAGTCACTATAGGAAGATGGCGAAACATCTTTACTACGG---- 56
BlkCd22         CTGCAGAATTCTAATACGAGTCACTATAGGAAGATGGCGAAACATCTTAATGGT-TT--- 56
BlkCd30         CTGCAGAATTCTAATACGAGTCACTATAGGAAGATGGCGAAACATCTTTACTAG-GA--- 56
BlkCd09         CTGCAGAATTCTAATACGAGTCACTATAGGAAGATGGCGAAACATCTTTACTAAGGC--- 57
BlkCd24         CTGCAGAATTCTAATACGAGTCACTATAGGAAGATGGCGAAACATCTTTACTAAGGATAG 60
BlkCd36         CTGCAGAATTCTAATACGAGTCACTATAGGAAGATGGCGAAACATCTTTACTAATG---- 56
BlkCd06         CTGCAGAATTCTAATACGAGTCACTATAGGAAGATGGCGAAACATCTTTACTAATAA--- 57
BlkCd11         CTGCAGAATTCTAATACGAGTCACTATAGGAAGATGGCGAAACATCTTTACT-------- 52
BlkCd14         CTGCAGAATTCTAATACGAGTCACTATAGGAAGATGGCGAAACATCTTTGCTG--A---- 54
BlkCd39         CTGCAGAATTCTAATACGAGTCACTATAGGAAGATGGCGAAACATCTTTACAAGAA---- 56
BlkCd12         CTGCAGAATTCTAATACGAGTCACTATAGGAAGATGGCGAAACATCTTTACTA-ACT--- 56
BlkCd38         CTGCAGAATTCTAATACGAGTCACTATAGGAAGATGGCGAAACATCTTTAGTA-ACA--- 56
BlkCd33         CTGCAGAATTCTAATACGAGTCACTATAGGAAGATGGCGAAACATCTTTACTA-TCC--- 56
BlkCd40         CTGCAGAATTCTAATACGAGTCACTATAGGAAGATGGCGAAACATCTTTACTAGTAA--- 57
BlkCd20         CTGCAGAATTCTAATACGAGTCACTATAGGAAGATGGCGAAACACCTTTAGTAGCAC--- 57
BlkCd25         CTGCAGAATTCTAATACGAGTCACTATAGGAAGATGGCGAAACATCTTTACTAACA---- 56
BlkCd32         CTGCAGAATTCTAATACGAGTCACTATAGGAAGATGGCGAAACATCTTTACTA------- 53
BlkCd35         CTGCAGAATTCTAATACGAGTCACTATAGGAAGATGGCGAAACATCTTTACTA------- 53
BlkCd28         CTGCAGAATTCTAATACGAGTCACTATAGGAAGATGGCGAAACACCTTTACTAGTTT--- 57
BlkCd05         CTGCAGAATTCTAATACGAGTCACTATAGGAAGATGGCGAAACATCTTTACAAGAT---- 56
BlkCd17         CTGCAGAATTCTAATACGAGTCACTATAGGAA-ATGGCGAAACATCTTTAGTAGTTGTAC 59
BlkCd04         CTGCAGAATTCTAATACGAGTCACTATAGGAAGATGGCGAAACATCTTTAGTAGTTT--- 57
BlkCd13         CTGCAGAATTCTAATACGAGTCACTATAGGAAGATGGCGAAACATCTTTAGT--TAT--- 55
BlkCd08         CTGCAGAATTCTAATACGAGTCACTATAGGAAGATGGCGAAACATCTTTAGTAGT----- 55
BlkCd34         CTGCAGAAT-----------TCGCCCTTGGAAGATGGCGAAACATCTTTAGTAATGG--- 46
                *********           **.* .*:**** ***********  **

BlkCd07         ----ATAGTTGAAATAGGTACAAGTATCACGGTGATATTG--TATCATG-TTAGTGTC-G 106
BlkCd26         ----ATAGTTGAAATAGGTACGAGTATCACGGTGATATTG--TATCATG-TTAGTGAC-G 106
BlkCd03         ----CGA-TAGAAATAGCGACAAGTCTAGGTGTGATTTATGCTCTCTT--CTAGTGAC-G 106
BlkCd10         ----CTAATCACTCGAAGAAG---AGTGGCGAGGAGTAAGAATGTCGTG-ATGGTGAC-G 106
BlkCd31         ----AAAGCAAAGCATAGAGCTCTACGGGTTAGGGGTACGAG-GTCGT---TGGTGAC-G 106
BlkCd18         ----ATACGGACAAAGAGTGG-CAGACAGAAACCT-TCGATAGCTC-AA-ATAGTGAC-G 106
BlkCd23         ----ATAAAACCAAAATTTGTTAAGACAGTGACCT-TCGATAGCAC-AA-ATAGTGAC-G 106
BlkCd19         ----ATACTTATAAATGGTCAATGAGCAATGTTCAGTAGCTTGTGCGAC-TTAGTGAC-G 106
BlkCd16         ----CCACTGATAGAGCTCATATTGGAAAGGAATA-TTGGTTATGAACC-CTAGTTAC-G 106
BlkCd01         ---TTGAAATA-CTTTTGGATT--TATATTATCATATGGCCGGGAGAT----AGTGAC-G 106
BlkCd27         ---GTGCCGCATCGTTAGGATT--ACGATAAGAACACCG-CGGGAAAT----AGTGAC-G 106
BlkCd15         ---GTA--ATGCCTCATGCTCTGTATATAATGAGGGGA-ACGTGAGGGTT----AGTGAC-G 106
BlkCd21         ---GTAGCACGCCGAAAGTGCT--ATACAGAGGGGAGTAGTGTGGGTT----AGTGAC-G 106
BlkCd02         ---AAACTACGTCGCGTGCATT--TAAAAGGCGAATAGAGAGGGGGTT----AGTGAC-G 106
BlkCd22         ---ACATAGTCCATCTAAGACC-TTGTCACCTTCATCGCGAAG-AAAT----AGTGAC-G 106
BlkCd30         ---GC-TAGGCCTCCTTATATT-TTATCACGAGTAGCTCACGGGAGAT----AGTGAC-G 106
BlkCd09         ---TCAATGAGCCGCGAAGGCG-GCAT-GCATACAAGTCTGGG-AGAT----AGTGAC-G 106
BlkCd24         TTAGTTGTAGGTTGACAACGTG--TGGGACTAGACT-------GGGTT----AGTGAC-G 106
BlkCd36         --TGTAGGAGACTCCCATCGTT--ATGGACATGTCTCTA-CATGGGGT----AGTCAC-G 106
```

```
BlkCd06         ---ATGGAGTGGTCAACCGAAT-TAGGAGCACGTAATG--CATGAGTT----AGTGAC-G 106
BlkCd11         --AGCAA-GTTTA-TTGCGGGT-TAATGACAGTTATAAAGCGTATCATTACTAGTGAC-G 106
BlkCd14         --AACATTGTTCAGTAGCCTTT-TATTAACACTAATAAAGAGCGACTT----AGTGAC-G 106
BlkCd39         --AGCAT-ATTCG-CAGCAGGG-GTATAGCGAAGATGAGTCGGAAGAT----AGTGACCG 107
BlkCd12         ----AACGGAAAGCAAGTTGAAGAATCGA---TTGTCGATTG--TGGGAGATAGTAAC-G 106
BlkCd38         ----AAC--ACACTAGGGTAAATATTGGG---ATTTCGAGTGGTTGCGCCTTAGTGAC-G 106
BlkCd33         ----GACGAGGAGCCCTGGGCCAGGG-GG---ATATCAGTTG-GAGGGAGATAGTGAC-G 106
BlkCd40         ----AAAGCCCAGCTATGGGTAGGTGTGG---GTATCCGTTT-ACGG--GATAGTGAC-G 106
BlkCd20         ----ATCAGGAGAAACGTGGAAAAGATAG---CTACTG--AATGGCGGCT-TAGTGAC-G 106
BlkCd25         ----ATCTGTATTATTGCTGTGGGGTGAG---TTACAG--AAGGTGGGAGGTAGTGAC-G 106
BlkCd32         ----AGCAAT--AGCAAAGGATTGCAGAATTTTCTATGGCTATCGCTGGGTTAGTGAC-G 106
BlkCd35         ----AGCAAT--AGCAAAGGATTGCAGAATTTTCTATGGCTATCGCTGGGTTAGTGAC-G 106
BlkCd28         ----AACAATGACACCAAAGAATGGAGGG---ACATTG--TTTCGCGGGG-TAGTGAC-G 106
BlkCd05         -----CAGGGGCGATAAGTCATAGAACAAAGGATCTCCTGATCGGGTT----AGTGAC-G 106
BlkCd17         T-TAACCGGGGACAGAAGACATAG-----AGGGTATC---AACGCCTT----AGTGAC-G 105
BlkCd04         ----AAACCGGAGTTGTCAATCAGA---CGTATGAAGGAAAAAC---GCCTTAGTGAC-G 106
BlkCd13         ----AAACCGGA-TTCACTAATAGG---CTGATACAGAGAGGGCTATGGGTTAGTGAC-G 106
BlkCd08         ----TCCAAGGGGATAAAAATGGAAGAGCGGGCCC-ATGGGAAC---GCCTTAGTGAC-G 106
BlkCd34         ----TCTCCGACGTCTAAACTGGGT---CGGACTAGAAGTTAAT---GCCTTAGTGAC-G 95
```

Table S6. Sequence alignment for the blocked selection with negative selection.

```
BN-Cd02      CTGCAGAATTCTAAT-ACGAGTCACTATAGGAAGATGGCGAAACA-TCTTA----GAGAT 54
BN-Cd20      CTGCAGAATTCTAAT-ACGAGTCACTATAGGAAGATGGCGAAACA-TCTTA----GCCAG 54
BN-Cd01      CTGCAGAATTCTAAT-ACGAGTCACTATAGGAAGATGGCGAAACA-TCTTTAATCGGTAA 58
BN-Cd10      CTGCAGAATTCTAAT-ACGAGTCACTATAGGAAGATGGCGAAACA-TCTTTAATCGGTAA 58
BN-Cd06      CTGCAGAATTCTAAT-ACGAGTCACTATAGGAAGATGGCGAAACA-CCTTTAATCGGTAA 58
BN-Cd30      CTGCAGAATTCTAAT-ACGAGTCACTATAGGAAGATGGCGAAACA-TCTTTAATCGGTAA 58
BN-Cd13      CTGCAGAATTCTAAT-ACGAGTCACTATAGGAAGATGGCGAAACA-CCTTG---CG-TAT 54
BN-Cd14      CTGCAGAATTCTAAT-ACGAGTCACTATAGGAAGATGGCGAAACA-CCTTG---CG-TAT 54
BN-Cd38      CTGCAGAATTCTAAT-ACGAGTCACTATAGGAAGATGGCGAAACA-TATTG---CT-CAT 54
BN-Cd04      CTGCAGAATTCTAAT-ACGAGTCACTATAGG-AGATGGCGAAACA-TTTAG---GGGTCC 54
BN-Cd21      CTGCAGAATTCTAAT-ACGAGTCACTATAGGAAGATGGCGAAACA-TCTTG---GGGTCT 55
BN-Cd40      CTGCAGAATTCTAAT-A-GAGTCACTATAGGAAGATGGCGAAACA-TCTTG---GGGCAC 54
BN-Cd07      CTGCAGAATTCTAAT-ACGAGTCACTATAGGAAGATGGCGAAACA-TCTTGCATAAATGT 58
BN-Cd35      CTGCAGAATTCTAAT-ACGAGTCACTATAGGAAGATGGCGAAACA-TCTTGCATAAATGT 58
BN-Cd12      CTGCAGAATTCTAAT-ACGAGTCACTATAGGAAGATGGCGAAACA-TCTTGCATAAATGT 58
BN-Cd09      CTGCAGAATTCTAAT-ACGAGTCACTATAGGAAGATGGCGAAACA-TCTTAG--ACGGCA 56
BN-Cd34      CTGCAGAATTCTAAT-ACGAGTCACTATAGGAAGATGGCGAAACA-TCTTAG--ACGGCA 56
BN-Cd24      CTGCAGAATTCTAAT-ACGAGTCACTATAGGAAGATGGCGAAACA-TCTTAG--ACGGCA 56
BN-Cd25      CTGCAGAATTCTAAT-ACSAGTCACTATAGGAAGATGGCGAAACA-TCTTAA--ACCAGG 56
BN-Cd36      CTGCAGAATTCTAAT-ACSAGTCACTATAGGAAGATGGCGAAACA-TCTTATG-AGCTAA 57
BN-Cd17      CTGCAGAATTCTAAT-ACGAGTCACTATAGGAAGATGGCGAAACA-TCTTTTTATTAAAA 58
BN-Cd23      CTGCAGAATTCTAAT-ACGAGTCACTATAGGAAGATGGCGAAACA-TCTTTTTATTAAAA 58
BN-Cd11      CTGCAGAATTCTAAT-ACGAGTCACTATAGGAAGATGGCGAAACA-TCTTTTTATTTAAA 58
BN-Cd03      CTGCAGAATTCTAAT-ACGAGTCACTATAGGAAGATGGCGAAACACCTTACACTCGAAGA 59
BN-Cd37      CTGCAGAATTCTAAT-ACGAGTCACTATAGGAAGATGGCGAAACACCTTACACTCGAAGA 59
BN-Cd05      CTGCAGAATTCTAAT-ACGAGTCACTATAGGAAGATGGCGAAACAATCTTCATTCGATAG 59
BN-Cd08      CTGCAGAATTCTAAT-ACGAGTCACTATAGGAAGATGGCGAAACA-TCTTCATTCGATAG 58
BN-Cd15      CTGCAGAATTCTAATAACGAGTCACTATAGGAAGATGGCGAAACA-TCTTCATTCGATAG 59
BN-Cd29      CTGCAGAATTCTAAT-ACGAGTCACTATAGGAAGATGGCGAAACA-CCTTCATTCGATAG 58
BN-Cd19      CTGCAGAATTCTAAT-ACGAGTCACTATAGGAAGATGGCGAAACA-CCTTCATTCGATAG 58
BN-Cd33      CTGCAGAATTCTAAT-ACGAGTCACTATAGGAAGATGGCGAAACA-CCTTCATTCGATAG 58
BN-Cd26      CTGCAGAATTCTAAT-ACGAGTCACTATAGGAAGATGGCGAAACA-TCTTCATTCGATAG 58
BN-Cd16      CTGCAGAATTCTAAT-ACGAGTCACTATAGGAAGATGGCGAAACA-TCTTTCTTCGATAG 58
BN-Cd22      CTGCAGAATTCTAAT-ACGAGTCACTATAGGAAGATGGCGAAACA-TCTTTCTTCGATAG 58
BN-Cd32      CTGCAGAATTCTAAT-ACGAGTCACTATAGGAAGATGGCGAAACA-TCTTTAC-CCAAAA 57
BN-Cd39      CTGCAGAATTCTAAT-ACGAGTCACTATAGGAAGATGGCGAAACA-CCTTTAATCCAAAC 58
BN-Cd18      CTGCAGAATTCTAAT-ACGAGTCACTATAGGAAGATGGCGAAACA-TCTTTACCCAAAAG 58
                ************** * .************ *************   *:
```

```
BN-Cd02       CTATTGAACGATAACTAATTAGCCATATTTATCCACCTACATCTTAGTGACGGTAAGCTT 114
BN-Cd20       CTGAAACAATCGAAGAGTTTTGCATATCGTGATGACGCAAAGAGTAGTGACGGTAAGCTT 114
BN-Cd01       CAGCAACAATAA-TAGGTTTCTACTGCTACG---TAGGGCCAATTAGTGACGGTAAGCTT 114
BN-Cd10       CAGCAACAATAA-TAGGTTTCTACTGCTACG---TAGGGCCAATTAGTGACGGTAAGCTT 114
BN-Cd06       CAGCGACAATAA-TAAGTTTGTACTGCTACG---TAGGGCCAATTAGTGACGGTAAGCTT 114
BN-Cd30       CAGCAACAATAA-TAAGTTTGTACTGCTACG---TAGGGCCAATTAGTGACGGTAAGCTT 114
BN-Cd13       CATCTTCAATTCGATAGAGTCCACGTCTACAGGAATGTGGGAAATAGTAACGGTAAGCTT 114
BN-Cd14       CATCTTCAATTCGATAGAGTCCACGTCTACAGGAATGTGGGAAATAGTAACGGTAAGCTT 114
BN-Cd38       YATCYTCAATTCGATAGAGTCCACGTTCACAAGAATGTGGGAAATAGTGACGGTAAGCTT 114
BN-Cd04       ATATTGCTCAAAGATAGT-TCGAACATCTGAAACGCACGAAGAATAGTGACGGTAAGCTT 113
BN-Cd21       ATATTGCTCAAAGATAGT-TCGTACAACTGAAACGCACGAAGAATAATGACGGTAAGCTT 114
BN-Cd40       GCTCTTAACCAAGATAATGTTAAGTATCTTACAGGAAC-CACTTTAGTGACGGTAAGCTT 113
BN-Cd07       CTACATGCAGAATATCCGCCA--TTTCATTCG--ACAGTAGAGATAGTGACGGTAAGCTT 114
BN-Cd35       CTACATGCAGAATATCCGCCA--TTTCATTCG--ACAGTAGAGATAGTGACGGTAAGCTT 114
BN-Cd12       CTACATGCAGAATATCCACCA--TTTCATTCG--ACAGTAGAGATAGTGACGGTAAGCTT 114
BN-Cd09       CCTGAGATGATTTAATCGCAGTTCTTCCTTCG--ATAGCTAAGATAGTGACGGTAAGCTT 114
BN-Cd34       CCTGAGATGATTTAATCGCAGTTCTTCCTTCG--ATAGCTAAGATAGTGACGGTAAGCTT 114
BN-Cd24       CCTGAGCTGATATAATCGCACTTCTTCCTTCG--ATAGCTAAGATAGTGACGGTAAGCTT 114
BN-Cd25       TGTCTTACTTTCTAAGCTGTCATCTTCATTCG--ATAGCACAAATAGTGACGGTAAGCTT 114
BN-Cd36       CGTAAAAGTTTCTAAAGCCAC-TGTTCCTTCS--ATAGTACAGATAGTGACGGTAAGCTT 114
BN-Cd17       CGTGTAAAAATGTGGGGGCAG--TTTCCTTCG--ATAGCCCAGATAGTGACGGTAAGCTT 114
BN-Cd23       CGTGTAAAAATGTAGGGGCAG--TTTCCTTCG--ATAGCCCAGATAGTGACGGTAAGCTT 114
BN-Cd11       CGTGTAAAAATGTAGGGGCAT--CTTCCTTCG--ACAGCCCAGATAGTGACGGTAAGCTT 114
BN-Cd03       GTCTCTTAAA--TTATACCTT--GTAGAATCCCCTG-GAGGAAATAGTGACGGTAAGCTT 114
BN-Cd37       GTCTCTTAAA--TTGTACCCG--GTACAATCCCCTG-GAGGAAATAGTGACGGTAAGCTT 114
BN-Cd05       TTGAAATAGG--TACGAGTAT--CACGGCGATGTTGTATCATGTTAGTAACGGTAAGCTT 115
BN-Cd08       TTGAAATAGG--TACAAGTAT--CACGGTGATATTGTATCACGTTAGTAACGGTAAGCTT 114
BN-Cd15       TTGAGATAGG--TACGAGTAT--CACGGTCATATTGTATCATGTTGGTGACGGTAAGCTT 115
BN-Cd29       TTGAGATAGG--TACAAGTAT--CACGGTGACATTGTATCATGTTAGTGTCGGTAAGCTT 114
BN-Cd19       TTAAAATAGG--TACGAGTAT--CACGGTGATATTGTACCATGTTAGTGACGGTAAGCTT 114
BN-Cd33       TTAAAATTGG--GAGGACATG--TAGTGGGACGATTCAACCCCCTAGTGACGGTAAGCTT 114
BN-Cd26       CTCAAATAGGACTATGTGTTT--AACTGGAATATG--AAAGGACTAGTGACGGTAAGCTT 114
BN-Cd16       TTAAAATAGT--GACTTCTAT--ATTAAGTCGCCTCATTGTTGATAGTGACGGTAAGCTT 114
BN-Cd22       TTAAGATAGT--GACTTCTAT--ATTAAGTCGTCTCATTGTTAATAATGACGGTAAGCTT 114
BN-Cd32       GGAG-TTCTTACGGATCCTAC--AATGAGGAAAAGTATGAGTTATAGTGACGGTAAGCTT 114
BN-Cd39       CACGGTGGTGACTGGCCTGAA--TAAGAGCATTAGACT--ATATTAGTGACGGTAAGCTT 114
BN-Cd18       GAAGGTTTTCTAATAACTGGC--TTTAGTAGAAACACA--GGAGTAGTGACGGTAAGCTT 114
```

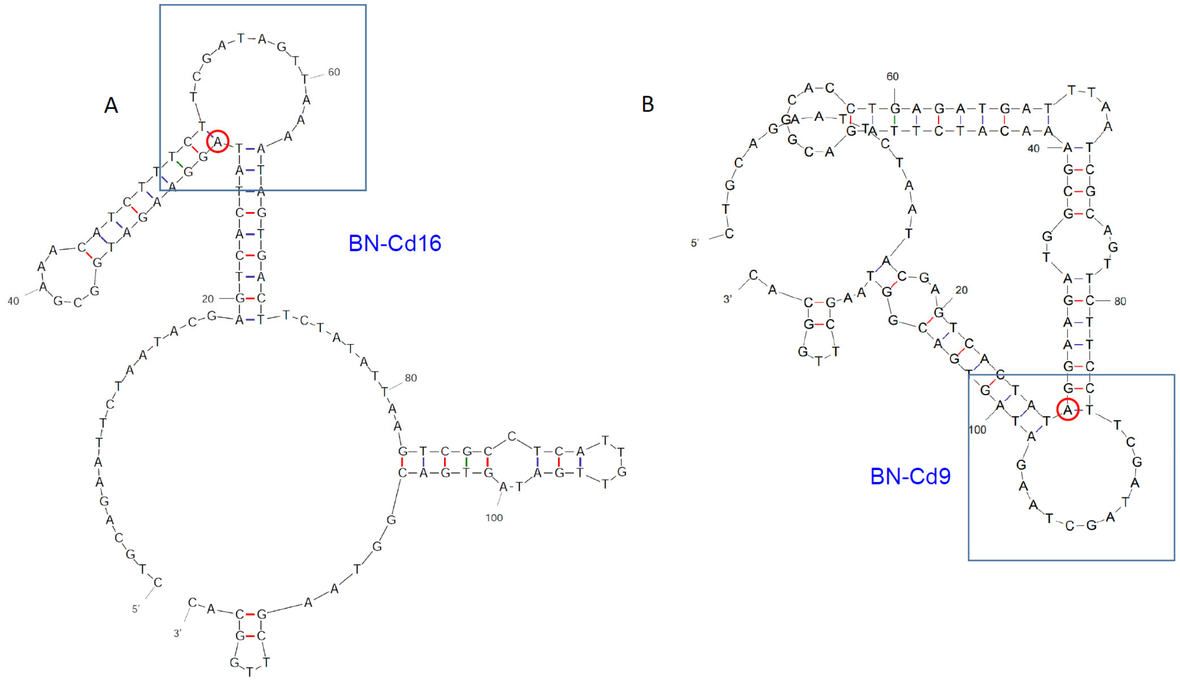

Figure S2. The M-fold predicted secondary structure of (A) BN-Cd16 and (B) BN-Cd9. The cleavage site ribo-adenine is marked in the red circle. A trans-cleaving version of BN-Cd16 is in Figure 2C of the main paper, where the redundant nucleotides on both ends were removed. Also, the loop around nucleotide 40 was opened. Outside the enzyme core region, the distribution of the redundant nucleotides is quite diverse, indicating that these redundant nucleotides do not play a role in catalysis.

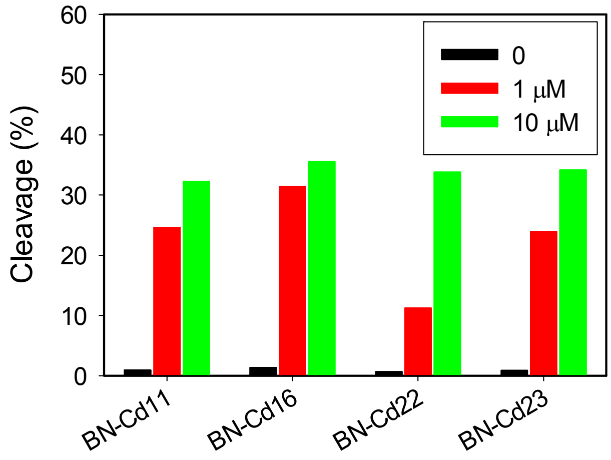

Figure S3. Gel-based assay of cleavage of the PS-modified and FAM labeled substrate (named PS-Sub in Table S2) by a few different enzymes (see Table S2 for sequences) in the presence of 1 μM and 10 μM Cd^{2+}. They all showed similar activity, which is consistent with their similar sequences in Figure 2D. BN-Cd16 was chosen for most of studies in this work. The reaction was in 50 mM MES, pH 6.0 with 25 mM NaCl after 1 h incubation time.

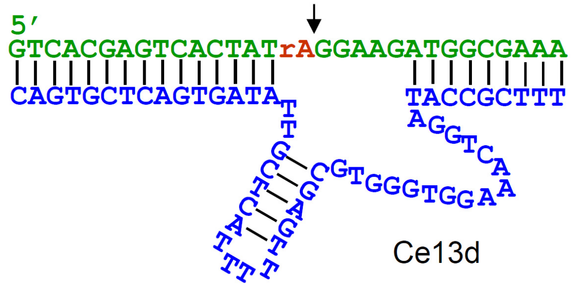

Figure S4. The structure of the Ce13d DNAzyme used in this study for comparison. The 3′-end of the substrate is labeled with a FAM fluorophore for its kinetic studies.

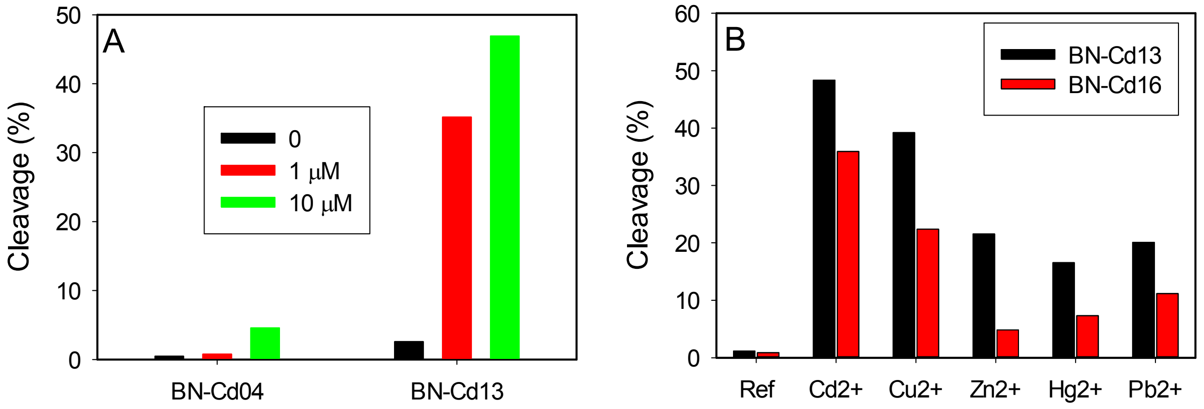

Figure S5. Additional gel-based assays on other DNAzymes using the PS-Sub. There are a few other types of DNAzyme sequences from our blocked negative selection. For example, BN-Cd13, BN-Cd14, and BN-Cd38 belong to the same family and we tested BN-Cd13 (A, the bars on the right side). It is quite active and cleaved nearly 50% with 10 μM Cd^{2+} in 1 h. Another DNAzyme, BN-Cd04 (and BN-Cd21) failed to show high activity and was not studied further. We next compared metal selectivity between BN-Cd13 and BN-Cd16 in (B). It appears that BN-Cd16 has higher selectivity (1 h reaction with 10 μM various metal ions). Each metal ion was used at 10 μM concentration with 1 h incubation in 50 mM MES, pH 6.0 with 25 mM NaCl.

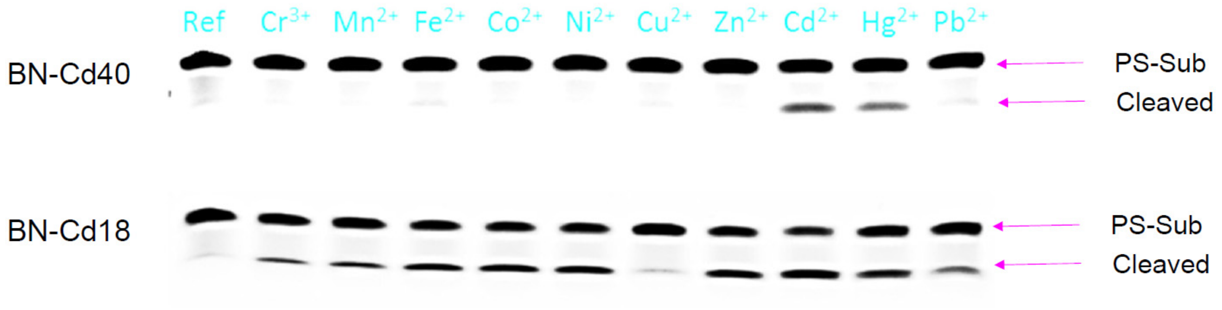

Figure S6. Metal specificity assay of the BN-Cd40 and BN-Cd18 DNAzymes using PS-Sub. BN-Cd40 has very good selectivity towards Cd^{2+} and this DNAzyme only appeared once in the blocked negative selection. BN-Cd18, on the other hand, has poor selectivity. Each metal ion was used at 10 µM concentration with 1 h incubation in 50 mM MES, pH 6.0 with 25 mM NaCl.

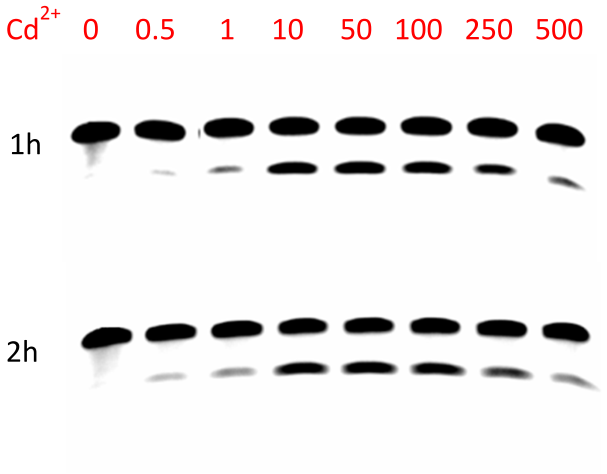

Cd^{2+} Concentration (uM)	% of Cleavage 1h	% of Cleavage 2h
0	0	0
0.5	2.3	4.9
1	2.9	7.6
10	21.9	28.0
50	23.9	30.4
100	17.5	26.8
250	8.9	15.4
500	5.0	9.9

Figure S7. Cd^{2+} concentration dependent study of the BN-Cd40 DNAzyme. Since the BN-Cd40 DNAzyme showed excellent metal specificity from the assay in Figure S6, it might also be suitable to be developed into a cadmium sensor. A range of Cd^{2+} concentrations (0-500 µM) was tested for 1 and 2 h. Both the gel images and quantifications are shown. With BN-Cd16, ~30% cleavage was achieved in 1 h, but BN-Cd40 only achieved ~3% cleavage under the same condition. Therefore, this is a much slower enzyme.

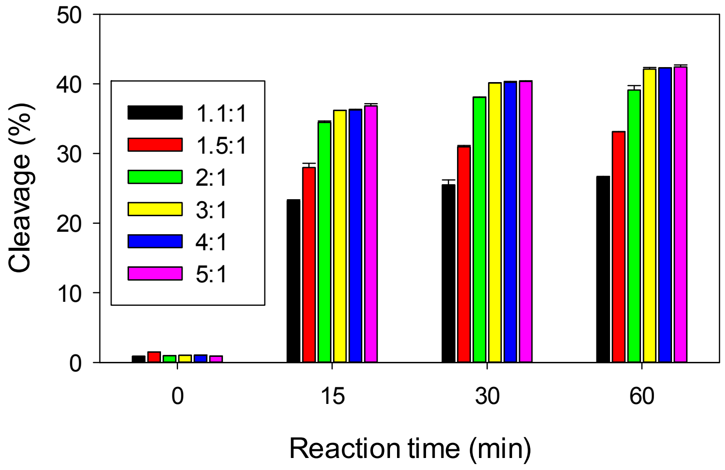

Figure S8. Quantification of PS-Sub cleavage as a function of BN-Cd16 enzyme concentration. At the high enzyme concentration (e.g. enzyme:substrate = 5:1), and after 1 h reaction with 10 µM Cd^{2+}, the cleavage of the substrate reached ~45%. The improvement from 30 min to 1 h was minimal for these samples as well. This suggests that only about half of the substrate can be cleaved by Cd^{2+}.

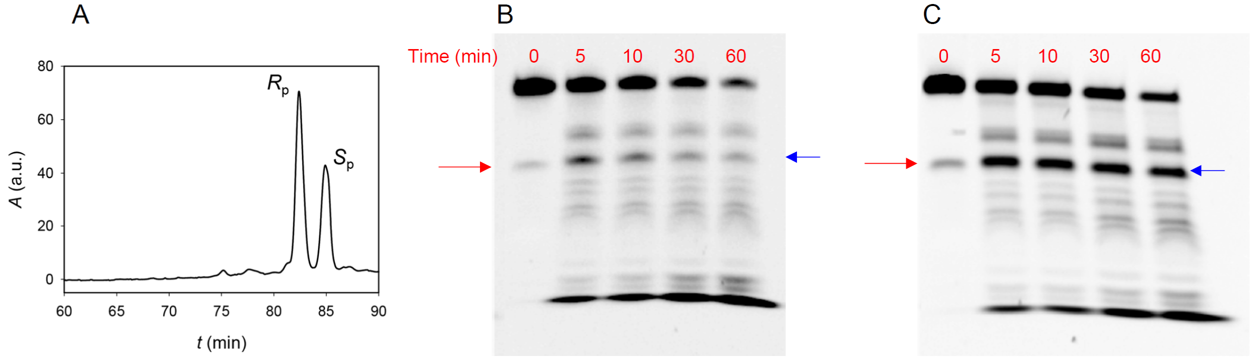

Figure S9. (A) HPLC trace of the two separated fragments. Snake venom digestion experiment to assign the HPLC separated fragments for the R_p (B) and S_p (C) as indicated in (A). 50 pmol of ligated PS substrate was diluted in 50 µL buffer (100 mM Tris, pH 8.5 and 2 mM $MgCl_2$). 1 µL of 0.01 unit/mL venom phosphodiesterase I was added and incubated at 37 °C up to an hour using Bio-Rad T-100 PCR instrument. Fraction of the samples were taken out and quenched with 1× gel loading buffer each time at designated time point. Digested samples were then separated in 15% denaturing PAGE gel at 200V for an hour. Gel image was taken with Bio-Rad ChemiDoc imaging system. The earlier fraction is digested more quickly and the later fraction is resistant to digestion, especially at the band indicated by the blue arrowhead, which corresponds to the rA*G junction position. The enzyme stops at the position. The red arrow indicates the cleavage product, which runs slightly faster than the ban indicated by the blue arrow, suggesting that these two bands differ by a single nucleotide. Therefore, the fraction in (B) is assigned as R_p and in (C) as S_p.

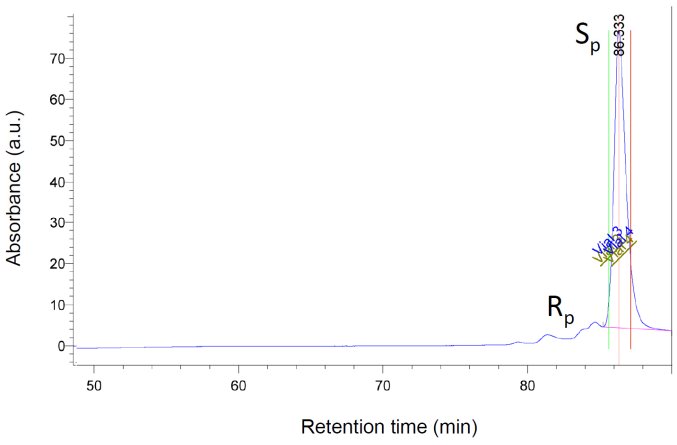

Figure S10. The re-purification of the S_p fragment (the S_p fragment from the first purification was re-injected). A small peak corresponding to R_p is observed and the collected S_p fractions are between the red and green lines, further increasing the purity.

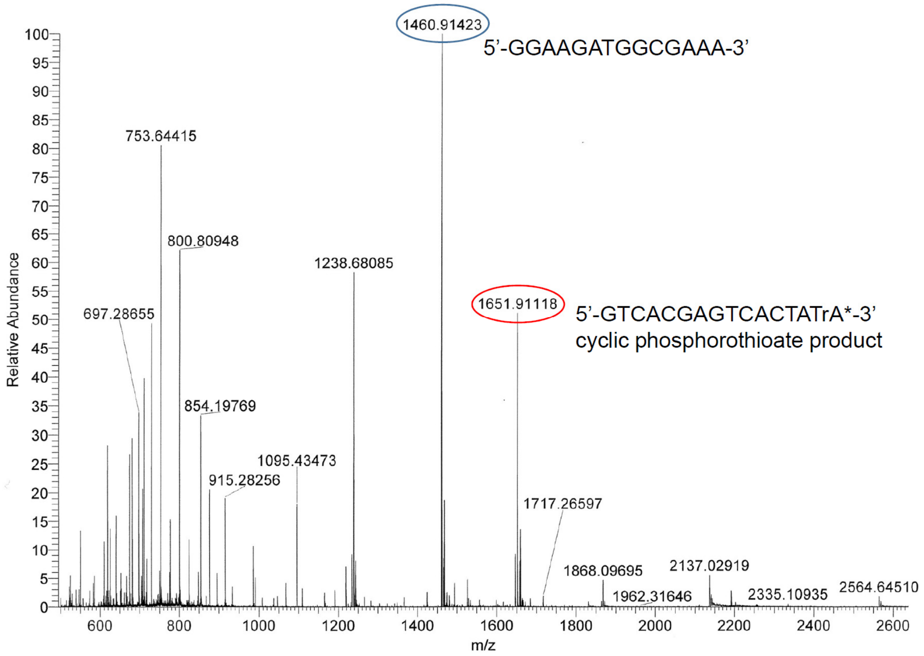

Figure S11. Mass spectrometry characterization of the BN-Cd16 cleavage product. The substrate sequence is 5′-GTCACGAGTCACTATrA*GGAAGATGGCGAAA. The calculated MW of the cyclic phosphorothioate product is 4959.22. The MW of the other fragment bearing 5′-hydroxyl group is 4385.9. The two highlighted peaks are the two fragments carrying three negative charges. These samples were prepared by reacting a non-labeled DNAzyme PS substrate with BN-Cd16 DNAzymes at 1 µM substrate and 1.5 µM enzyme concentration in 50 mM MES buffer (pH 6) with 25 mM NaCl and 10µM Cd^{2+}. Then the samples were desalted using a Sep-Pak column and dried. After rehydration in water to ~20 µM substrate concentration, the samples were analyzed using an ESI mass spectrometer.

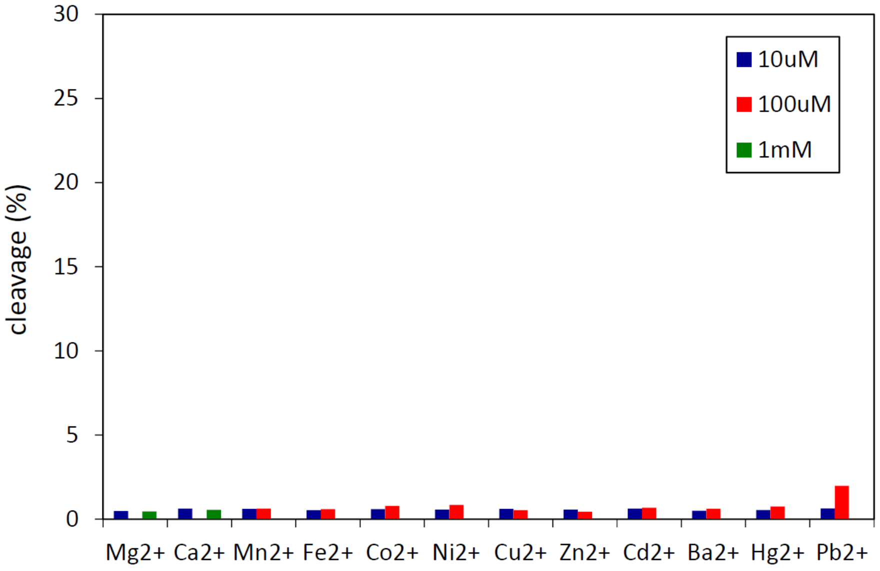

Figure S12. Cleavage of the PO substrate (PO-Sub) by BN-Cd16 in the presence of different metal ions. Only 2% cleavage was observed with a high concentration of Pb^{2+} in 1 h and others only showed only background signal. Therefore, this DNAzyme is highly specific for the PS substrate.

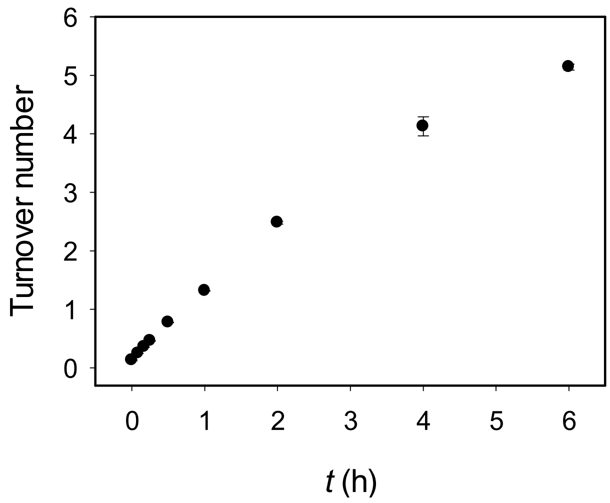

Figure S13. Analysis of the BN-Cd16 DNAzyme in the presence of 0.2 μM Cd^{2+}. The DNAzyme concentration was 5 μM. Therefore, to completely cleave the DNAzyme, each Cd^{2+} needs to turnover 25 DNAzyme. In this experiment, we observed ~5 cleavage events for each Cd^{2+} in 6 h, corresponding to ~50 min for each cleavage. To observe the turning over effect of Cd^{2+}, we only used 0.2 μM Cd^{2+}. Faster turnover is possible by increasing Cd^{2+} to its optimal concentration of 10 μM.

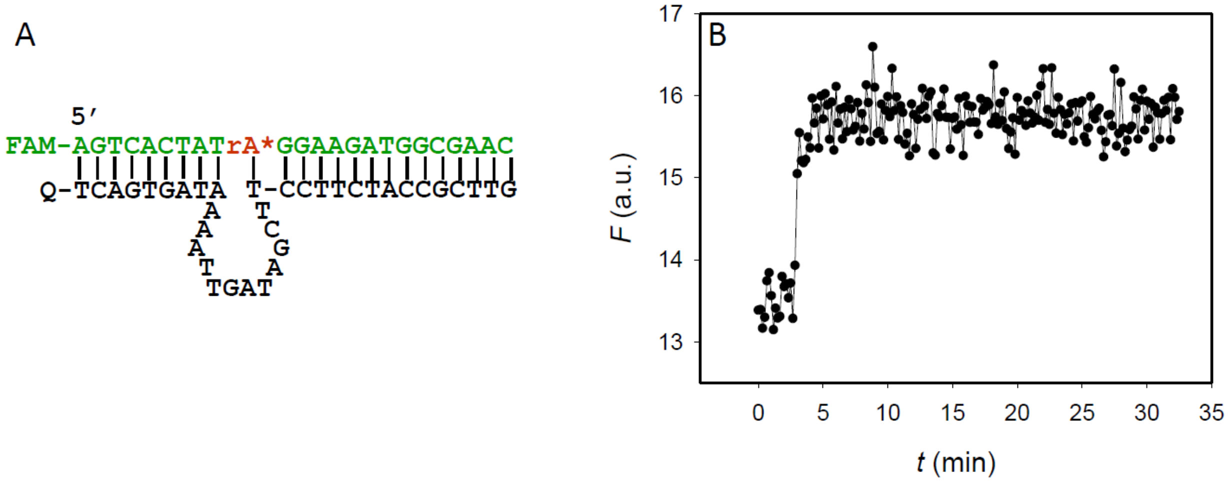

Figure S14. (A) The DNAzyme sequence for the fluorescent sensor design. (B) Sensor response to 100 nM Hg^{2+} and only an initial increase was observed after adding Hg^{2+} at 3 min. This response can be eliminated by filtering out the data in the first minute.

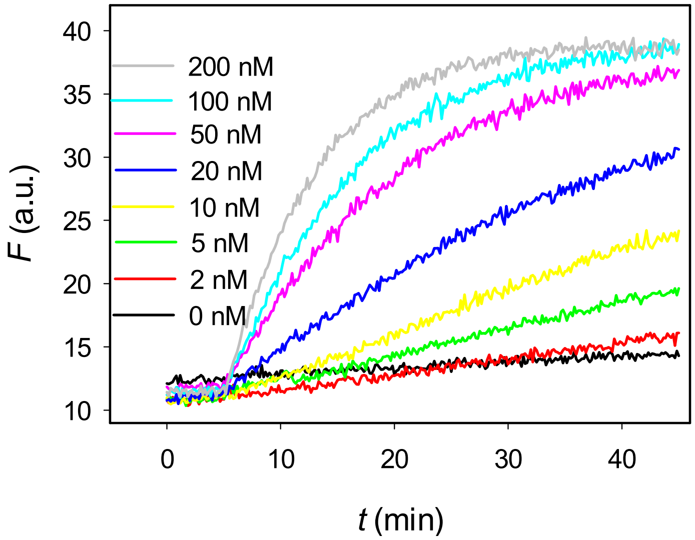

Figure S15. Sensor response using the untreated PS substrate containing an equal amount of each diastereomer. The reaction conditions are identical to that in Figure 6A of the main paper, and various concentrations of Cd^{2+} was added at 5 min. The signal increase was only ~3.5-fold as compared to the ~6-fold for the substrate after the 17E DNAzyme and Mg^{2+} treatment.
